# Identification of Genes Potentially Regulated by Human Polynucleotide Phosphorylase (*hPNPase^old-35^*) Using Melanoma as a Model

**DOI:** 10.1371/journal.pone.0076284

**Published:** 2013-10-15

**Authors:** Upneet K. Sokhi, Manny D. Bacolod, Santanu Dasgupta, Luni Emdad, Swadesh K. Das, Catherine I. Dumur, Michael F. Miles, Devanand Sarkar, Paul B. Fisher

**Affiliations:** 1 Department of Human and Molecular Genetics, Virginia Commonwealth University, Richmond, Virginia, United States of America; 2 VCU Institute of Molecular Medicine, Virginia Commonwealth University, Richmond, Virginia, United States of America; 3 VCU Massey Cancer Center, Virginia Commonwealth University, Richmond, Virginia, United States of America; 4 Department of Pathology, Virginia Commonwealth University, Richmond, Virginia, United States of America; 5 Department of Pharmacology and Toxicology, Virginia Commonwealth University, Richmond, Virginia, United States of America; 6 Department of Neurology, Virginia Commonwealth University, Richmond, Virginia, United States of America; The University of Tennessee Health Science Center, United States of America

## Abstract

Human Polynucleotide Phosphorylase (*hPNPase^old-35^* or *PNPT1*) is an evolutionarily conserved 3′→5′ exoribonuclease implicated in the regulation of numerous physiological processes including maintenance of mitochondrial homeostasis, mtRNA import and aging-associated inflammation. From an RNase perspective, little is known about the RNA or miRNA species it targets for degradation or whose expression it regulates; except for *c-myc* and miR-221. To further elucidate the functional implications of *hPNPase^old-35^* in cellular physiology, we knocked-down and overexpressed *hPNPase^old-35^* in human melanoma cells and performed gene expression analyses to identify differentially expressed transcripts. Ingenuity Pathway Analysis indicated that knockdown of *hPNPase^old-35^* resulted in significant gene expression changes associated with mitochondrial dysfunction and cholesterol biosynthesis; whereas overexpression of *hPNPase^old-35^* caused global changes in cell-cycle related functions. Additionally, comparative gene expression analyses between our *hPNPase^old-35^* knockdown and overexpression datasets allowed us to identify 77 potential “*direct”* and 61 potential “*indirect”* targets of *hPNPase^old-35^* which formed correlated networks enriched for cell-cycle and wound healing functional association, respectively. These results provide a comprehensive database of genes responsive to *hPNPase^old-35^* expression levels; along with the identification new potential candidate genes offering fresh insight into cellular pathways regulated by PNPT1 and which may be used in the future for possible therapeutic intervention in mitochondrial- or inflammation-associated disease phenotypes.

## Introduction

Ribonucleases (RNases) are one of the central players involved in the regulation of post-transcriptional control of gene expression in both prokaryotes and eukaryotes [1], [2]. They are divided into two main categories, endo- and exo-ribonucleases. Depending on the direction of degradation, exoribonucleases can be further classified as 5′→3′ or 3′→5′ exoribonucleases [3]. Numerous exoribonucleases identified in bacteria, Archaea and Eukarya have been placed under six major superfamilies, RBN, RNR, DEDD, PDX, RRP4 and 5PX [4], [5]. Of these, the PDX family is the only one whose members use inorganic phosphate to generate nucleotide diphosphates instead of hydrolytic cleavage [5]. Polynucleotide phosphorylase (PNPase) is an evolutionarily conserved phosphorolytic 3′→5′ exoribonuclease that belongs to the PDX family of proteins [4] and it plays a major role in RNA metabolism in bacteria, plants and humans. The protein encoded by this gene consists of five conserved classical domains: two RNase PH domains, a α–helical domain and two RNA binding domains KH and S1 [6]. The human homolog of this gene (*hPNPase^old-35^*) was identified in an overlapping pathway screen (OPS) intended to identify upregulated transcripts in terminally differentiated human melanoma cells and senescent progeroid fibroblasts [7].

Human polynucleotide phosphorylase (hPNPase^old-35^) is encoded by the *PNPT1* gene mapping to chromosome 2p15-2p16.1 and has been characterized as a type I IFN (IFN-α/β)-inducible early response gene [7], [8]. Numerous endeavors over the past decade have enriched our comprehension of the workings of this exoribonuclease. It has become increasingly clear over the years that the various physiological functions of this enzymatic protein are not restricted to a single cellular compartment, in this case the mitochondrial inter membrane space (IMS) where it is primarily located [9], [10]. In the cytoplasm this protein performs a myriad of functions, which include but are not restricted to degradation of mRNA and miRNA species [11], [12]. Adenoviral-mediated overexpression of hPNPase^old-35^ causes growth inhibition of normal and cancer cells characterized by morphological changes associated with senescence, G_1_/S or G_2_/M cell-cycle arrest and apoptosis [11], [13], [14], [15]. The ability of hPNPase^old-35^ to selectively degrade *c-myc* mRNA through its exoribonucleolytic activity has been identified as a key molecular mechanism mediating the growth suppressive effects of hPNPase^old-35^, since overexpression of *c-myc* could only partially rescue these effects [11]. Apart from mRNA degradation, hPNPase^old-35^ has also been identified as a direct regulator of mature miRNA species, specifically miR-221 that targets the cell cycle inhibitor p27^Kip1^ [12], [16]. Both these specialized functions of hPNPase^old-35^ also implicate it as a direct mediator of IFN-β-induced growth inhibition [12], [17]. Another novel function of hPNPase^old-35^ is its ability to generate double-stranded RNA (dsRNA) through a currently unknown mechanism, which ultimately leads to apoptosis of cells due to the activation of dsRNA-dependent protein kinase (PKR) [13].

Apart from the above-mentioned cytoplasmic functions that focus on its physiological roles in the regulation of growth inhibition and senescence, numerous roles of *hPNPase^old-35^* have been revealed that are central to its location in the mitochondria [16], [18], [19], [20]. Overexpression of hPNPase^old-35^ induces reactive oxygen species (ROS) production in the mitochondria resulting in the expression of pro-inflammatory cytokines, which is a major phenomenon linking hPNPase^old-35^ to aging-related inflammation [21], [22]. The significance of *hPNPase^old-35^* in maintaining mitochondrial homeostasis, with a specific relevance to the electron transport chain (ETC) components, has been revealed by *hPNPase^old-35^* knockdown studies in cell systems and also in a liver-specific knockout mouse model of *hPNPase^old-35^* [9], [23]. There are also studies providing evidence that *hPNPase^old-35^* is a regulator of mitochondrial RNA import and plays a role in mtRNA processing [23]-[27]. Recently hPNPase^old-35^ has also been shown to be present in the nucleus, and is associated with nEGFR protein, which regulates the exoribonuclease activity of hPNPase^old-35^ upon exposure to ionizing radiations [28].

Most of what we know about *hPNPase^old-35^* today has been gleaned from classical gain-of-function or loss-of-function experiments and from comparative studies performed through lessons acquired from its bacterial and plant counterparts [16], [19], [20]. Although these studies have been immensely valuable in illuminating the importance of *hPNPase^old-35^* in various physiological phenomena like senescence, growth-inhibition and mitochondrial dynamics, little is known about the specific network of genes that are involved in these processes or which might be dysregulated when *hPNPase^old-35^* is aberrantly expressed. Moreover, when evaluating its function as an exoribonuclease, we know of only one mRNA and a single miRNA species that hPNPase^old-35^ can degrade directly, *c-myc* and miR-221, respectively. Such findings led to our interest in trying to identify genes or gene networks that are either potential direct degradation targets of hPNPase^old-35^ or regulated by *hPNPase^old-35^*, respectively. In order to pursue this objective, we performed gene expression analysis on human melanoma cells in which *hPNPase^old-35^* was either silenced or ectopically overexpressed. Studying the global gene expression changes associated with *hPNPase^old-35^*-knockdown or overexpression has provided valuable new insights regarding the functions of this elusive exoribonuclease while also validating previously known information. With the help of Ingenuity Pathway Analysis (IPA) we have identified key biological functions and associated genes that are deregulated in response to aberrant expression of *hPNPase^old-35^*. Additionally, comparative analysis of the knockdown and overexpression datasets has allowed us to identify novel genes that may be directly or indirectly regulated by *hPNPase^old-35^*.

In summary, our present studies interrogated the global implications of *hPNPase^old-35^* dysregulation and now provide a comprehensive database that can be further used not only to understand the biological functions of *hPNPase^old-35^* but also to identify candidate direct degradation targets such as *c-myc* and miR-221.

## Materials and Methods

### Cell culture

The human melanoma cell line HO-1 [29]–[31] was initially provided by Dr. Eliezer Huberman (Argonne National Laboratories, IL) maintained in Dulbecco's Modified Eagle Medium (DMEM; Invitrogen Life Technologies) supplemented with 10% fetal bovine serum (FBS; Sigma) and 5% penicillin/streptomycin (Gibco). The melanoma cell line WM35 [32]–[35], provided by Dr. Meenhard Herlyn (Wistar Institute) was maintained in MCDB153:Leibovitz's L15 (4∶1) pH 7.4 supplemented with 2% FBS and CaCl_2_ (1.68 mM, Sigma). The *hPNPase^old-35^*-knockdown stable cell lines were maintained in growth medium as described with the addition of 200 ng/ml (in case of HO-1 cells) or 300 ng/ml (in case of WM35 cells) of the selective antibiotic puromycin. The melanoma cell lines C8161 and MeWo were cultured as previously described [11], [12]. All cell lines were maintained in a 5% CO_2_ 95% O_2_ humidified incubator at 37°C. 0.5% Trypsin-EDTA (10X) solution was purchased from Gibco and 1X Dulbecco's Phosphate-Buffered Saline (DPBS) from Corning Cellgro.

### Expression constructs, stable cell lines and viral infections

Lentiviral constructs (pGIPZ) expressing GFP were purchased from Open Biosystems. The constructs contained non-silencing short hairpin RNA (shRNA) or shRNAs against *hPNPase^old-35^* (PNPshRNA-1: clone ID V2LHS_17644, Mature Sense: GGCAACAGGAAATTAGAAA, Mature Antisense: TTTCTAATTTCCTGTTGCC; PNPshRNA-2: clone ID V2LHS_159887, Mature Sense: CAATAGGATTGGTCACCAA, Mature Antisense: TTGGTGACCAATCCTATTG). Lentiviruses encoding the different shRNAs were produced by cotransfecting the HEK-293T packaging cells with the appropriate pGIPZ constructs and the Trans-lentiviral Packaging Mix (Open Biosystems) according to the manufacturer's protocol. The supernatants containing the lentiviral particles were harvested 48 hours after transfection, concentrated by centrifugation and frozen at −80°C as aliquots. 1ml of the above viral suspension was used to transduce 3×10^5^ HO-1 human melanoma cells grown in 6-cm dishes supplemented with 10 µg/ml polybrene. 48 hours post-transduction cells were trysinized and replated at a low density (1∶5) and complete media was added supplemented with positive selection marker puromycin to establish stable shRNA expressing single clones over a period of two weeks. Single clones isolated for PNPshRNA-1 and PNPshRNA-2 were screened to assess the level of knockdown at both the RNA and protein levels and the HO-1 clones with maximum *hPNPase^old-35^* knockdown were used for microarray analyses (we used one of our HO-1 PNPshRNA-1 clones for this purpose).

The construction and purification of replication-incompetent adenovirus encoding *hPNPase^old-35^* (Ad.*hPNPase^old-35^*) has been described previously [7], [11]. The empty vector Ad.*vec* was used as a control. For all adenoviral experiments, 1×10^5^ cells were grown in 6-cm dishes and infected after 24 hours with Ad.*vec* or Ad.*hPNPase^old-35^* at a final m.o.i. of 5000 vp/cell diluted in 1 ml of serum-free media. After 6 hours of infection with shaking every 15 minutes, complete media was added and cells were harvested at the required time points (e.g., 36 hours post-infection) for microarray analysis, RNA or protein isolation.

### RNA extraction, quality assessment and Microarray analyses

Total RNA from the *hPNPase^old-35^*-knockdown cell line (HO-1 melanoma cells stably expressing shRNA-1 against *hPNPase^old-35^*), HO-1 cells expressing non-silencing control shRNA, and HO-1 cells infected with Ad.*hPNPase^old-35^* or Ad.*vec* for 36 hours was isolated from cell lysates in TRIZOL reagent (InvitrogenTM Life Technologies, Carlsbad, CA). Cell lysates were subjected to an automated extraction method using the MagMAX™-96 for Microarrays Total RNA Isolation Kit (Ambion/InvitrogenTM Life Technologies, Carlsbad, CA) on the MagMAX™ Express Magnetic Particle Processor (Applied Biosystems/InvitrogenTM LifeTechnologies, Carlsbad, CA).

Gene expression profiles were ascertained using GeneChip® Human Genome U133A 2.0 (HG-U133A 2.0) arrays (Affymetrix, Santa Clara, CA) as previously described. Every chip was scanned at a high resolution, with pixelations ranging from 2.5 µm down to 0.51 µm, by the Affymetrix GeneChip® Scanner 3000 according to the GeneChip® Expression Analysis Technical Manual procedures (Affymetrix, Santa Clara, CA). After scanning, the raw intensities for every probe were stored in electronic files (in.DAT and.CEL formats) by the GeneChip® Operating Software (GCOS) (Affymetrix, Santa Clara, CA). The overall quality of each array was assessed by monitoring the 3′/5′ ratios for a housekeeping gene (GAPDH) and the percentage of “Present” genes (%P); where arrays exhibiting GAPDH 3′/5′<3.0 and %P>40% were considered good quality arrays [36]. All experiments were done in biological triplicates. The microarray data generated for this study are available online at the Gene Expression Omnibus repository under the accession number GSE46884.

### Statistical Analysis, IPA and functional classification of genes

The Robust Multiarray Average method (RMA) was used for normalization and generating probe set expression summaries for the gene expression assays. To identify genes significantly altered among the different conditions (i.e., *hPNPase^old-35^* down-regulation and up-regulation), t-tests were performed for each cell type. To adjust for multiple hypothesis testing, the resulting p-values were used to obtain the false discovery rates using the q-value method. Genes were considered significant using an FDR of 5%. All analyses were performed in the R statistical environment using functions provided by the BioConductor packages [36], [37].

In order to categorize biological functions related to gene expression altered by *hPNPase^old-35^* in our microarray analyses, we used the Ingenuity Pathway Analysis (IPA, Ingenuity® Systems, http://www.ingenuity.com) [38], [39]. Genes were considered differentially expressed if they had q-values ≤0.05. The Affymetrix probe set IDs of significantly altered genes identified through the statistical analysis described above in both the *hPNPase^old-35^*-knockdown and overexpression scenarios, along with their associated p- and q-values were uploaded into IPA and analyses performed.

The ToppFun function of the ToppGene suite of web applications [40] was used for the functional enrichment of the *hPNPase^old-35^*-directly and indirectly regulated genes. The HGNC symbols for both the gene lists were uploaded and a FDR correction threshold of 5% was set for the subsequent functional enrichment analyses. The Gene Ontology (GO) categories (molecular function, biological process, and cellular component), biological pathway and gene and miRNA families were considered for analyses.

In order to further validate if genes regulated by alterations in *hPNPase^old-35^* expression formed gene interaction networks, the gene symbols for the potential *hPNPase^old-35^*-“*directly*” and -“*indirectly*” regulated genes were uploaded into GeneMANIA (http://www.genemania.org), a web interface for generating interactive functional association networks [41]. This resource utilizes multiple external datasets, including protein-protein interactions and published microarray datasets, to form networks of potential gene-gene interactions. The interactive functional association networks obtained were generated based on co-expression, biological pathways, predicted association, genetic interactions, physical interactions and co-localization functional association data. The networks were generated using the query-dependent automatically selected weighting method.

### cDNA synthesis and quantitative real-time RT-PCR (qRT-PCR)

Total RNA was harvested from the *hPNPase^old-35^*-knockdown and control stable cell lines and from the adenovirus infected HO-1 cells using the RNeasy purification kit (Qiagen). The quality and concentrations of isolated RNA samples were assessed using the NanoDrop 2000 (Thermo Scientific). 2 µg of RNA was used in a total volume of 20 µl to synthesize cDNA using the High Capacity cDNA Reverse Transcription kit (Applied Biosystems) according to the manufacturer's instructions. Real-time quantitative PCR was conducted using the ViiA™ 7 Real-Time PCR System (Applied Biosystems) and performed in a total volume of 20 µl that contained the TaqMan Gene Expression Master Mix (Applied Biosystems), 1 µl of the cDNA template generated and the target-specific TaqMan Gene expression assays (Applied Biosystems) according to following cycle parameters: 95°C for 10 minutes followed by 40 cycles at 95°C for 15 seconds and at 60°C for 1 minute. Each sample was run in triplicate using three biological replicates and normalized to the housekeeping gene GAPDH used as an internal control in each case. The ΔΔC_t_ method was used for comparing relative fold expression differences in the genes of interest between different test samples.

### Protein isolation and Western blot analysis

Cells were harvested by centrifugation, pellets washed in PBS and subsequently lysed in ice-cold 1X cell lysis buffer (Cell Signaling) supplemented with PhosSTOP Phosphatase Inhibitor Cocktail Tablets and complete Mini Protease Inhibitor Cocktail Tablets (Roche), followed by centrifugation at 13,000 rpm for 15 minutes at 4°C. The supernatant or whole cell lysate was collected in a fresh tube and protein concentration was measured using the Bio-Rad Protein Assay Dye Reagent Concentrate (BIO-RAD). 30 µg of total cell lysate was mixed with SDS sample buffer and heated for 5 minutes at 95°C. The proteins were separated by 8–10% SDS-PAGE gels and transferred onto nitrocellulose membranes and blocked using 5% non-fat milk supplemented with 1% bovine serum albumin (BSA) in TBS-T for 1 hour. After washing three times with TBS-T for 10 minutes each, the membranes were incubated with primary antibodies overnight at 4°C. The primary antibodies used were anti-*hPNPase^old-35^* (chicken; 1∶5000), anti-EF1α (mouse, 1∶1000). The next day membranes were washed as before and incubated with the relevant horseradish-peroxidase conjugated secondary antibodies for 1 hour at room temperature. After washing three times with TBS-T for 10 minutes each, the proteins were detected using ECL Western Blotting detection reagent (GE Healthcare Life Sciences) and exposed to X-ray film.

## Results

### Melanoma cell culture model for studying *hPNPase^old-35^* regulated gene expression

**Figure 1:**
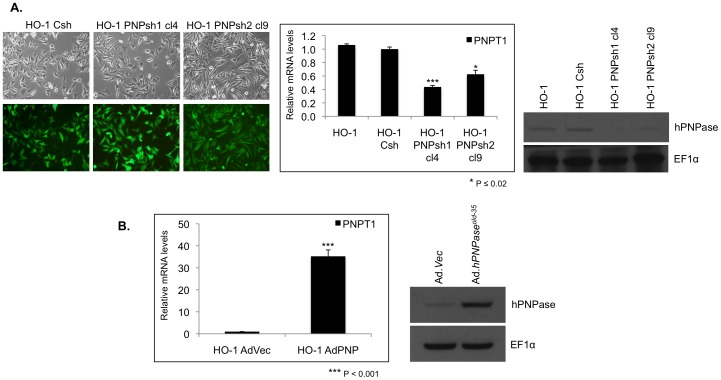
Generation of a melanoma cell culture model for hPNPase^old-35^ expression. (A) Phase contrast LM (top) and GFP fluorescent micrographs (bottom) of HO-1 melanoma cell lines following transduction with GFP expressing scrambled shRNA (HO-1 Csh) and *hPNPase^old-35^* shRNA1 (shown in clone 4; cl4) and 2 (shown is clone 9; cl9) expressing lentiviruses and selection with puromycin. qRT-PCR expression of *hPNPase^old-35^* (*hPNPase^old-35^* knockdown) normalized to control (shScramble). Mean values normalized to a GAPDH internal reference; error bars represent mean ± S.E. of three replicate experiments. Anti-hPNPase^old-35^ and EF1α loading control immunoblots. (B) qRT-PCR expression of *hPNPase^old-35^* in HO-1 cells infected with Ad.*hPNPase^old-35^* normalized to cells infected with Ad.*Vec* for 36 h. Immunoblot showing hPNPase^old-35^ overexpression compared to Ad.*Vec* post 36 hour of infection. Error bars represent mean ± S.E of three replicate experiments. * P<0.02, *** P<0.001.

In order to establish stable HO-1 human melanoma cell lines in which *hPNPase^old-35^* expression was silenced, we employed a lentiviral system to ensure efficient delivery. RNA and protein levels of *hPNPase^old-35^* were analyzed in the different single clones that survived after puromycin selection as described. Both the shRNAs tested, PNPshRNA-1 (data shown for clone 4) and PNPshRNA-2 (data shown for clone 9), resulted in ≥60% knockdown at the protein level (Figure 1A) relative to the scrambled control shRNA. PNPshRNA-1 clone 4 was used for microarray analysis.

The replication-deficient adenovirus for ectopic overexpression of *hPNPase^old-35^* has been extensively characterized previously [7], [11]. Infection of HO-1 cells was performed as previously described [7], [11] and both RNA and protein were analyzed for overexpression of *hPNPase^old-35^* (Figure 1B). RNA from these cells was collected 36 h post-infection for microarray analysis.

### Genetic profile of *hPNPase^old-35^*-knockdown melanoma cells

**Figure 2:**
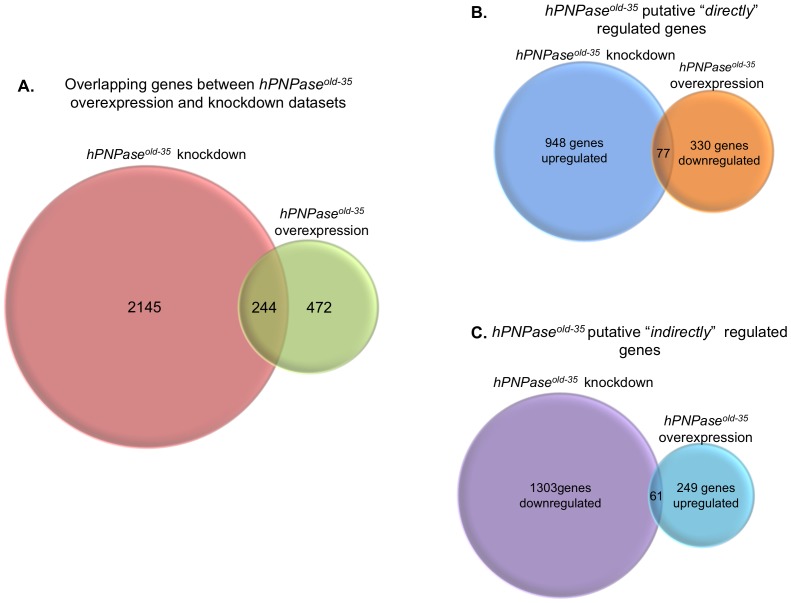
Venn diagrams representing number of genes significantly altered when *hPNPase^old-35^* is knocked down or overexpressed in human melanoma cells. Shown are total number of dysregulated genes (A), genes “*directly*” (B) and “*indirectly*” (C) regulated by *hPNPase^old-35^*.

In order to further understand and possibly identify novel functions of *hPNPase^old-35^*, we stably depleted it using shRNA in HO-1 human melanoma cells (Figure 1A). The ability to study changes in gene expression patterns has become a valuable technique that permits evaluation of the significance of a gene in a more global context. Microarray analysis between the *hPNPase^old-35^* shRNA and the scrambled shRNA expressing HO-1 cells led to the identification of a total of 1025 upregulated and 1364 downregulated transcripts which were altered significantly (FDR ≤0.05) (Figure 2).

**Figure 3:**
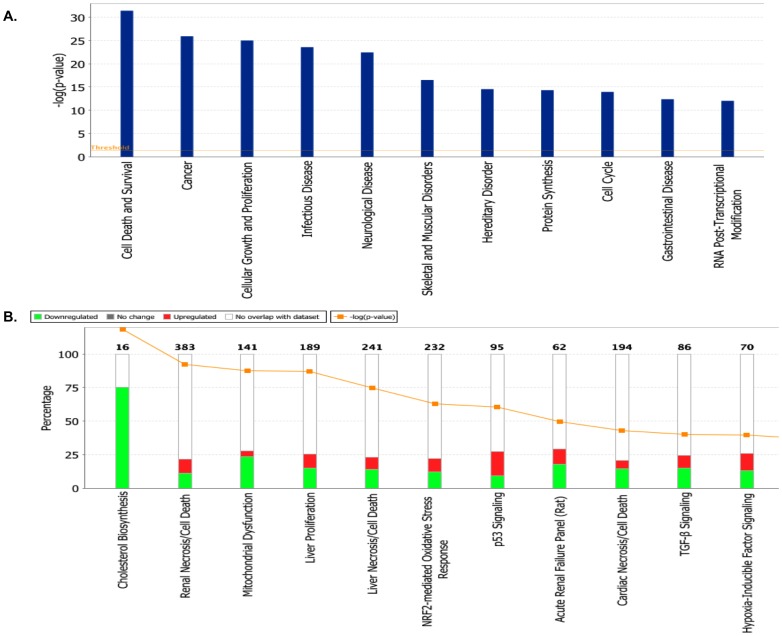
Functional analysis of genes dysregulated as a result of *hPNPase^old-35^* depletion. (A) The biological functions and states associated with genes differentially expressed when *hPNPase^old-35^* is knocked down in human melanoma cells. (B) Toxicologically related functionalities and pathways associated with genes dysregulated (proportions shown in graphs) after *hPNPase^old-35^* knockdown in melanoma cells, as identified by IPA Toxicogenomic Analysis.

We utilized IPA to functionally categorize all the differentially expressed genes identified following *hPNPase^old-35^* knockdown in HO-1 melanoma cells. Since the microarray experiments used to identify the dysregulated pathways or networks affected after *hPNPase^old-35^* depletion were based on a single shRNA, we cannot claim that all these changes are truly an effect of *hPNPase^old-35^* depletion as there is a chance that some of these may be off-target effects. Future studies based solely on the pathways or networks identified here will need to be validated using multiple shRNAs or *hPNPase^old-35^* wobble mutants. The main molecular and cellular functions (p-values ranging from 3.92E-32 - 1.26E-03) associated with the most significantly altered genes were cell death and survival, cellular growth and proliferation, protein synthesis, cell cycle and RNA post-translational modification (Figure 3A, Table S1A). Based on these functional categories 25 biological gene networks were generated by IPA with a score ranging from 41 to 28 (Table S1D). IPA computes scores for each network based on p-values, which in turn indicate the likelihood of genes being found together in a network due to random chance. Higher the score, lesser the chance of the genes in a network being grouped together by random chance alone.

Apart from classifying individual genes into functional categories, IPA also predicts corresponding biological pathways that may be significantly altered, along with mechanisms related to toxicity at a more biochemical level (Figure 3). The most significant canonical pathways identified were related to EIF2 signaling, cholesterol biosynthesis, integrin signaling and mitochondrial dysfunction (Figure S1A, Table S1C). Even more fascinating was the finding that two of these biological pathways, cholesterol biosynthesis (Table 1) and mitochondrial dysfunction (Tables 2 and 3), were directly correlated with predicted physiological toxicity (identified through IPA-Tox analysis) (Figure 3B, Table S1B). *HMGCR* (Figure S2A), *HMGCS1* and *IDI1* were the three most significantly altered genes belonging to the cholesterol biosynthesis pathway. An overall downregulation of genes belonging to the electron transport chain and some associated factors was observed, consistent with previous observations regarding the role of *hPNPase^old-35^* in maintaining mitochondrial homeostasis [9], [23]. Some of the significantly downregulated genes were *NDUFA3*, *NDUFS1*, *UQCRFS1*, *COX6B1*, *C0X7A1*, *ATP5C1*, *CAT* and *UCP2* (Figure S2B).

**Table 1:** List of genes which are significantly altered as a result of *hPNPase^old-35^* stable knockdown, and are associated with cholesterol biosynthesis, according to IPA Toxicogenomic Analysis.

Gene Symbol	Gene Name	Fold Change	Affymetrix ID
ACAT2	acetyl-CoA acetyltransferase 2	−1.29 *	209608_s_at
DHCR7	7-dehydrocholesterol reductase	−1.51 **	201790_s_at
EBP	emopamil binding protein (sterol isomerase)	−1.22 *	213787_s_at
FDFT1	farnesyl-diphosphate farnesyltransferase 1	−1.23 *	210950_s_at
FDPS	farnesyl diphosphate synthase	−1.17 *	201275_at
HMGCR	3-hydroxy-3-methylglutaryl-CoA reductase	−1.42 ***	202539_s_at
HMGCS1	3-hydroxy-3-methylglutaryl-CoA synthase 1 (soluble)	−1.56 ***	221750_at
IDI1	isopentenyl-diphosphate delta isomerase 1	−1.92 ***	208881_x_at
LSS	lanosterol synthase (2,3-oxidosqualene-lanosterol cyclase)	−1.29 *	202245_at
MVD	mevalonate (diphospho) decarboxylase	−1.24 *	203027_s_at
SC5DL	sterol-C5-desaturase (ERG3 delta-5-desaturase homolog, S. cerevisiae)-like	−1.26 *	211423_s_at
SQLE	squalene epoxidase	−1.37 **	209218_at

**Table 2:** List of ETC components which are significantly altered as a result of *hPNPase^old-35^* stable knockdown, and are associated with mitochondrial dysfunction, according to IPA Toxicogenomic Analysis.

ETC components	Gene symbol	Gene name	Fold change	Affymetrix ID
Complex I	NDUFA2	NADH dehydrogenase (ubiquinone) 1 alpha subcomplex, 2, 8 kDa	−1.11 *	209224_s_at
	NDUFA3	NADH dehydrogenase (ubiquinone) 1 alpha subcomplex, 3, 9 kDa	−1.32 ***	218563_at
	NDUFA8	NADH dehydrogenase (ubiquinone) 1 alpha subcomplex, 8, 19 kDa	−1.14 *	218160_at
	NDUFA13	NADH dehydrogenase (ubiquinone) 1 alpha subcomplex, 13	1.22 **	220864_s_at
	NDUFAB1	NADH dehydrogenase (ubiquinone) 1, alpha/beta subcomplex, 1, 8 kDa	−1.07 *	202077_at
	NDUFAF1	NADH dehydrogenase (ubiquinone) complex I, assembly factor 1	−1.09 *	204125_at
	NDUFB4	NADH dehydrogenase (ubiquinone) 1 beta subcomplex, 4, 15 kDa	−1.09 *	218226_s_at
	NDUFB5	NADH dehydrogenase (ubiquinone) 1 beta subcomplex, 5, 16 kDa	−1.09 *	203621_at
	NDUFB7	NADH dehydrogenase (ubiquinone) 1 beta subcomplex, 7, 18 kDa	1.36 **	202839_s_at
	NDUFS1	NADH dehydrogenase (ubiquinone) Fe-S protein 1, 75 kDa (NADH-coenzyme Q reductase)	−1.22 ***	203039_s_at
	NDUFS2	NADH dehydrogenase (ubiquinone) Fe-S protein 2, 49 kDa (NADH-coenzyme Q reductase)	−1.18 **	201966_at
	NDUFS3	NADH dehydrogenase (ubiquinone) Fe-S protein 3, 30 kDa (NADH-coenzyme Q reductase)	−1.10 *	201740_at
Complex II	SDHB	succinate dehydrogenase complex, subunit B, iron sulfur (Ip)	−1.11 *	202675_at
	SDHC	succinate dehydrogenase complex, subunit C, integral membrane protein, 15 kDa	−1.19 *	210131_x_at
Complex III	UQCR10	ubiquinol-cytochrome c reductase, complex III subunit X	−1.13 *	218190_s_at
	UQCR11	ubiquinol-cytochrome c reductase, complex III subunit XI	1.22 **	202090_s_at
	UQCRFS1	ubiquinol-cytochrome c reductase, Rieske iron-sulfur polypeptide 1	−1.32 ***	208909_at
Complex IV	COX6B1	cytochrome c oxidase subunit VIb polypeptide 1 (ubiquitous)	−1.31 ***	201441_at
Cytochrome c oxidase	COX7A1	cytochrome c oxidase subunit VIIa polypeptide 1 (muscle)	−1.80 ***	204570_at
	SURF1	surfeit 1	−1.17 **	204295_at
Complex V	ATP5A1	ATP synthase, H+ transporting, mitochondrial F1 complex, alpha subunit 1, cardiac muscle	−1.12 **	213738_s_at
ATP synthase	ATP5B	ATP synthase, H+ transporting, mitochondrial F1 complex, beta polypeptide	−1.10 *	201322_at
	ATP5C1	ATP synthase, H+ transporting, mitochondrial F1 complex, gamma polypeptide 1	−1.40 ***	213366_x_at

**Table 3:** List of mitochondria associated factors which are significantly altered as a result of *hPNPase^old-35^* stable knockdown, and are associated with mitochondrial dysfunction, according to IPA Toxicogenomic Analysis.

Gene symbol	Gene name	Fold change	Affymetrix ID
AIFM1	apoptosis-inducing factor, mitochondrion-associated, 1	−1.11 ***	205512_s_at
APH1B	anterior pharynx defective 1 homolog B (C. elegans)	−1.37 **	221036_s_at
BACE2	beta-site APP-cleaving enzyme 2	−1.11 *	217867_x_at
CAT	Catalase	−1.45 ***	201432_at
CYB5R3	cytochrome b5 reductase 3	−1.10 *	201885_s_at
GLRX2	glutaredoxin 2	−1.28 *	219933_at
GPX4	glutathione peroxidase 4	1.21 *	201106_at
GSR	glutathione reductase	−1.31 *	205770_at
HSD17B10	hydroxysteroid (17-beta) dehydrogenase 10	−1.13 *	202282_at
MAP2K4	mitogen-activated protein kinase kinase 4	1.44 *	203266_s_at
PDHA1	pyruvate dehydrogenase (lipoamide) alpha 1	−1.15 *	200980_s_at
PSEN2	presenilin 2 (Alzheimer disease 4)	−1.15 *	211373_s_at
PSENEN	presenilin enhancer 2 homolog (C. elegans)	−1.26 *	218302_at
SDHAP1	succinate dehydrogenase complex, subunit A, flavoprotein pseudogene 1	−1.19 **	222021_x_at
TRAK1	trafficking protein, kinesin binding 1	1.18 *	202080_s_at
UCP2	uncoupling protein 2 (mitochondrial, proton carrier)	−3.22 ***	208998_at

### Gene expression profile of Ad.*hPNPase^old-35^*-infected melanoma cells

Overexpression of *hPNPase^old-35^* causes growth inhibition in a number of cancer types and the growth prohibitive effects have been attributed to the downregulation of c-myc RNA by hPNPase^old-35^. We performed a microarray analysis of HO-1 cells infected with Ad.*hPNPase^old-35^* (Figure 1B) and compared the gene expression changes to cells infected with Ad.*vec* to identify transcripts differentially regulated as a result of *hPNPase^old-35^* overexpression. A total of 310 transcripts were upregulated and 407 were downregulated significantly (FDR≤0.05) (Figure 2).

**Figure 4:**
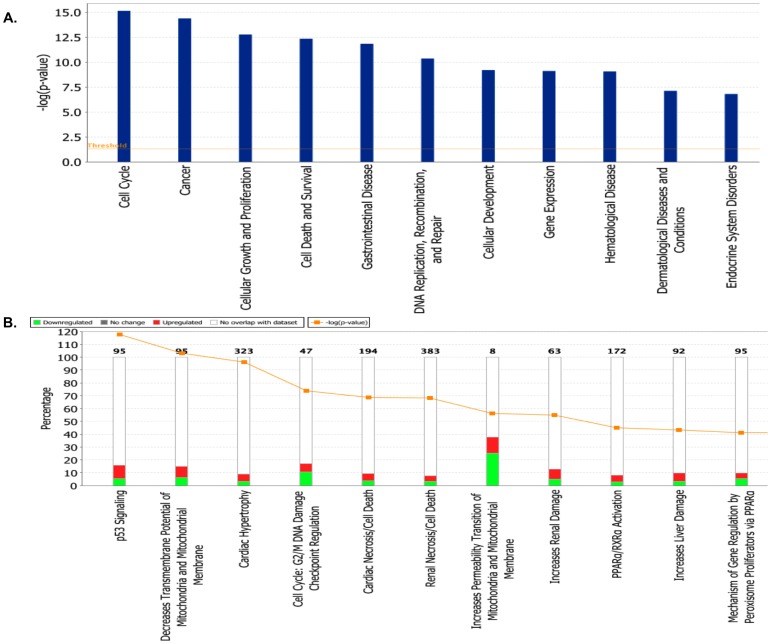
Functional analysis of genes dysregulated as a result of *hPNPase^old-35^* overexpression. (A) The biological functions and states associated with genes differentially expressed when *hPNPase^old-35^* is overexpressed in human melanoma cells. (B) Toxicologically related functionalities and pathways associated with genes dysregulated (proportions shown in graphs) after *hPNPase^old-35^* overexpression in melanoma cells, as identified by IPA Toxicogenomic Analysis.

Significant gene expression changes associated with adenoviral overexpression of *hPNPase^old-35^* could be classified into the following molecular and cellular functional categories according to IPA (p-values ranging from 7.50E-16 – 2.05E-03): cell cycle, cellular growth and proliferation, cell death and survival, DNA replication, recombination and repair and cellular development (Figure 4A, Table S2A). Based on these functional categories 25 biological gene networks were generated by IPA with a score ranging from 46 to 19 (Table S2D).

The following are the most significant biological pathways altered when *hPNPase^old-35^* was overexpressed: hereditary breast cancer signaling, p53 signaling, cell cycle control of chromosomal replication, IGF-1 and EIF2 signaling (Figure S1B, Table S2C). In order to understand if these pathways were relevant to any disease phenotype, we made use of the IPA-Tox analysis which identified p53 signaling, decreases Transmembrane Potential of Mitochondria and Mitochondrial Membrane (Table 4) and Cell Cycle: G2/M DNA Damage Checkpoint Regulation (Table 5) as some of the predicted significantly affected mechanisms (Figure 4B, Table S2B). The two main pathways we were interested to analyze further were the ones involved with cell cycle regulation and mitochondria, as there have been previous reports implicating a role for *hPNPase^old-35^* in both these cellular functions [9], [20]. Overexpression of *hPNPase^old-35^* causes growth inhibition, which is partially attributed to downregulation of *c-myc* [11], so it was not surprising to us when we observed changes in gene expression related to cell cycle regulation. Some of the most significantly altered genes in this category were *CCNB2*, *CDK1*, *CHEK2* and *KAT2B* (Table 5). Another interesting observation was the dysregulation of mitochondrial homeostasis, which again emphasizes a role of *hPNPase^old-35^* in the mitochondria. These gene expression changes could be due to a direct role that *hPNPase^old-35^* plays in the mitochondria or affects associated with growth inhibition caused by *hPNPase^old-35^* overexpression. Some of these significantly altered genes were *FAS*, *BCL2L11* and *BIRC5* (Table 4).

**Table 4:** List of genes which are significantly altered as a result of *hPNPase^old-35^* overexpression, and are functionally associated with the maintenance of mitochondrial transmembrane potential, according to IPA Toxicogenomic Analysis.

Gene Symbol	Gene Name	Fold Change	Affymetrix ID
APAF1	apoptotic peptidase activating factor 1	1.113**	211553_x_at
BARD1	BRCA1 associated RING domain 1	−1.414**	205345_at
BCL2L11	BCL2-like 11 (apoptosis facilitator)	1.351**	222343_at
BIRC5	baculoviral IAP repeat containing 5	−1.312**	202094_at
BNIP3	BCL2/adenovirus E1B 19 kDa interacting protein 3	−1.280**	201848_s_at
CD47	CD47 molecule	1.148**	213857_s_at
CHEK2	checkpoint kinase 2	−1.387**	210416_s_at
CHMP5	charged multivesicular body protein 5	1.715**	218085_at
FAS	Fas (TNF receptor superfamily, member 6)	1.450**	204781_s_at
IFI6	interferon, alpha-inducible protein 6	1.345**	204415_at
NFKB1	nuclear factor of kappa light polypeptide gene enhancer in B-cells 1	−1.404**	209239_at
SLC25A14	solute carrier family 25 (mitochondrial carrier, brain), member 14	−1.155**	204587_at
SOD2	superoxide dismutase 2, mitochondrial	1.384**	216841_s_at
TGM2	transglutaminase 2 (C polypeptide, protein-glutamine-gamma-glutamyltransferase)	1.423**	201042_at

**Table 5:** List of genes which are significantly altered as a result of *hPNPase^old-35^* overexpression, and are associated with G2/M DNA damage checkpoint regulation, according to IPA Toxicogenomic Analysis.

Gene Symbol	Gene Name	Fold Change	Affymetrix ID
BRCA1	breast cancer 1, early onset	−1.235**	211851_x_at
CCNB2	cyclin B2	−1.217**	202705_at
CDK1	cyclin-dependent kinase 1	−1.496**	203214_x_at
CHEK2	checkpoint kinase 2	−1.387**	210416_s_at
KAT2B	K(lysine) acetyltransferase 2B	1.520**	203845_at
SKP1/SKP1P2	S-phase kinase-associated protein 1	1.173**	207974_s_at
YWHAB	tyrosine 3-monooxygenase/tryptophan 5-monooxygenase activation protein, beta polypeptide	−1.139**	217717_s_at
YWHAZ	tyrosine 3-monooxygenase/tryptophan 5-monooxygenase activation protein, zeta polypeptide	1.332**	200639_s_at

### Identification of genes regulated by *hPNPase^old-35^*

Bearing in mind that *hPNPase^old-35^* is functionally an exoribonuclease, there could be two major mechanisms through which it could modulate gene expression; it could either degrade target genes directly (*“direct regulation”*) or it could degrade miRNAs (or mRNAs for other regulatory proteins) that in turn are regulators of certain genes (*“indirect regulation”*). Since we had gene expression patterns corresponding to both *hPNPase^old-35^* overexpression and knockdown, we employed stringent criteria to identify genes that could be directly or indirectly regulated by *hPNPase^old-35^* based on the hypothesis we proposed. Instead of making the obvious choice of selecting genes that were inversely related to the expression of *hPNPase^old-35^* in either microarray dataset, we performed an overlapping screen with the help of the online tool VENNY [42] in order to identify transcripts whose expressions changed inversely in both the datasets. This comparison resulted in the identification of 77 potential *hPNPase^old-35^*-directly regulated genes that were up-regulated when *hPNPase^old-35^* was depleted and down-regulated when *hPNPase^old-35^* was overexpressed (Figure 2B; Table S3A, B). A second set of 61 transcripts that were downregulated when *hPNPase^old-35^* was depleted by shRNA and up-regulated when *hPNPase^old-35^* was overexpressed were also taken in consideration as potential *hPNPase^old-35^*-indirectly regulated genes (Figure 2C; Table S3C, D). All the genes in these lists were significant with q-values ≤0.05.

**Figure 5:**
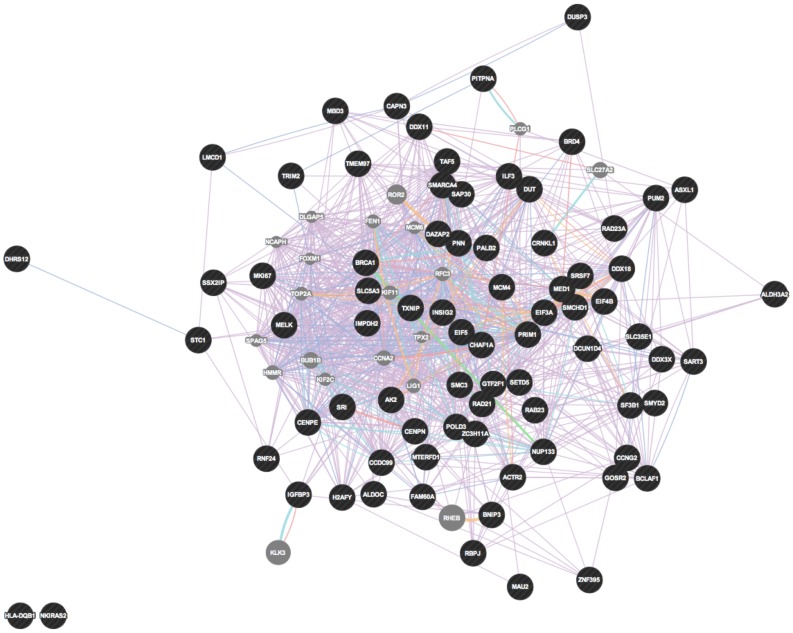
Network visualization of genes potentially “*directly*” regulated by *hPNPase^old-35^*.

**Figure 6:**
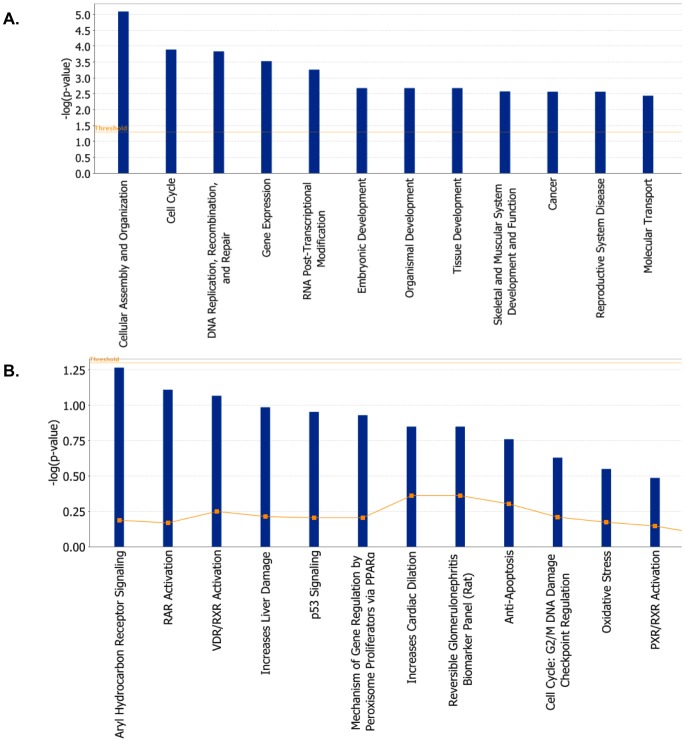
Functional analysis *hPNPase^old-35^*-putative “*directly*” regulated genes. (A) The biological functions and states associated with *hPNPase^old-35^*-putative “*directly*” regulated genes in human melanoma cells. (B) Toxicologically related functionalities and pathways associated with *hPNPase^old-35^*-putative “*directly*” regulated genes, as identified by IPA Toxicogenomic Analysis.

**Figure 7:**
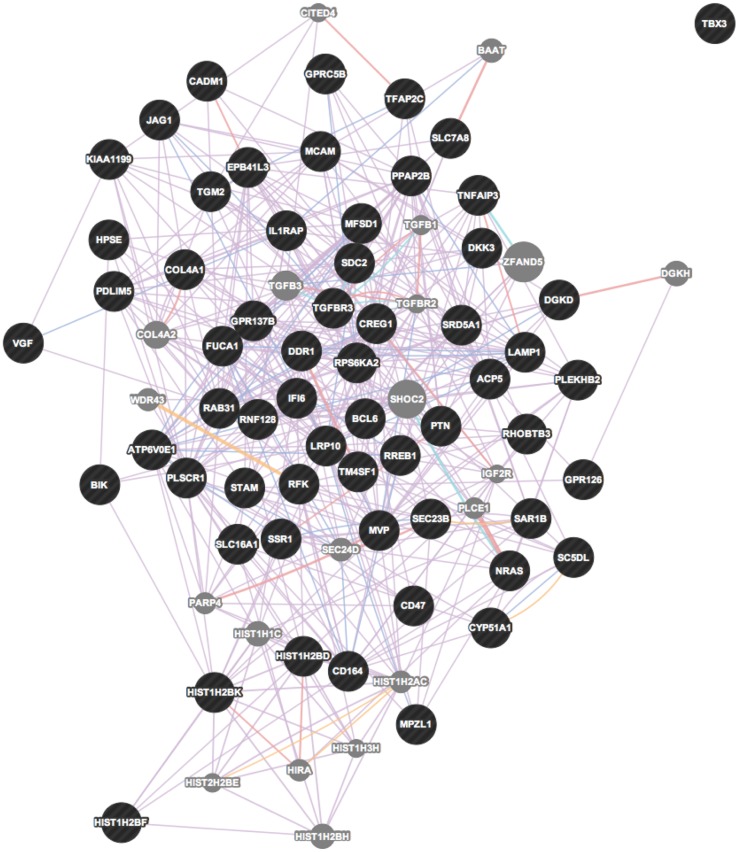
Network visualization of genes potentially “*indirectly*” regulated by *hPNPase^old-35^*.

**Figure 8:**
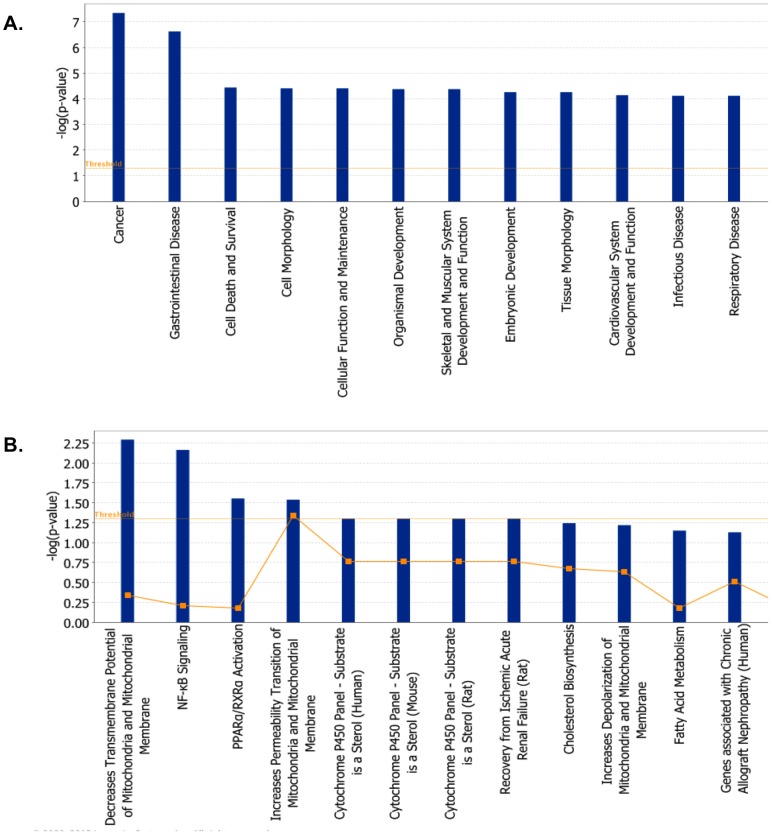
Functional analysis of *hPNPase^old-35^*-putative “*indirectly*” regulated genes. (A) The biological functions and states associated with *hPNPase^old-35^*-putative “*indirectly*” regulated genes in human melanoma cells. (B) Toxicologically related functionalities and pathways associated with *hPNPase^old-35^*-putative “*indirectly*” regulated genes, as identified by IPA Toxicogenomic Analysis.

In order to place the *hPNPase^old-35^*-regulated genes into functional categories, we made use of the ToppFun function of the ToppGene suite of web applications. A summary of the GO categories with the maximum number of genes and the most significant p-values is provided in Table 6. The *hPNPase^old-35^*-directly regulated genes represent significant enrichment related to RNA binding, chromosome organization and cell cycle associated (*CENPE*, *MKI67*, *POLD3*, *MCM4*) functions (Table S4A). To determine how the *hPNPase^old-35^*-regulated genes might interact with each other, gene symbols were uploaded into GeneMANIA. The directly regulated genes form a densely correlated network with overlapping functional categories akin to organelle fission, chromosome segregation and DNA strand elongation as predicted by the gene functional analysis (Figure 5). IPA analysis of this gene list also grouped the genes in similar biological categories (Figure 6). There was an over-representation of genes related to cellular response to wounding (*TGM2*, *SDC2*, *MCAM*), of genes belonging to the CD family of cell surface receptors (*DDR1*, *JAG1*, *CD164*, *MCAM*, *CD47*) and miR-124a predicted targets (*SLC7A8*, *JAG1*, *SDC2*, *CADM1*, *RNF128*) in the *hPNPase^old-35^*-indirectly regulated genes dataset (Table S4B). These probe sets also form a network cluster comprised mainly of co-expressed genes (Figure 7). IPA analysis of this gene list grouped the genes into cellular function and related categories (Figure 8). The inter-gene correlations between probe sets defined as being potentially directly or indirectly regulated by *hPNPase^old-35^* were largely based on publically available co-expression association data.

**Table 6:** Functional and structural categories of genes associated with *hPNPase^old-35^*-driven regulation.

Functional category	Source	p-value	no. of genes
**“** ***Directly*** **” regulated genes**			
RNA binding	GO: Molecular Function	6.21E-05	13/894
Chromosome organization	GO: Biological Process	1.28E-06	19/751
Cell cycle	GO: Biological Process	1.28E-06	25/1460
Chromosome	GO: Cellular Component	1.46E-10	21/671
Mitotic Prometaphase pathway	MSigDB	3.62E-02	6/92
**“** ***Indirectly*** **” regulated genes**			
Response to wounding	GO: Biological Process	5.43E-03	16/1168
Vacuole	GO: Cellular Component	4.74E-03	9/424
Integral to plasma membrane	GO: Cellular Component	4.81E-02	13/1328
CD molecules	Gene Family (genenames.org)	3.45E-04	5/276
miR-124a	MicroRNA (PicTar)	4.04E-02	11/626

**Figure 9:**
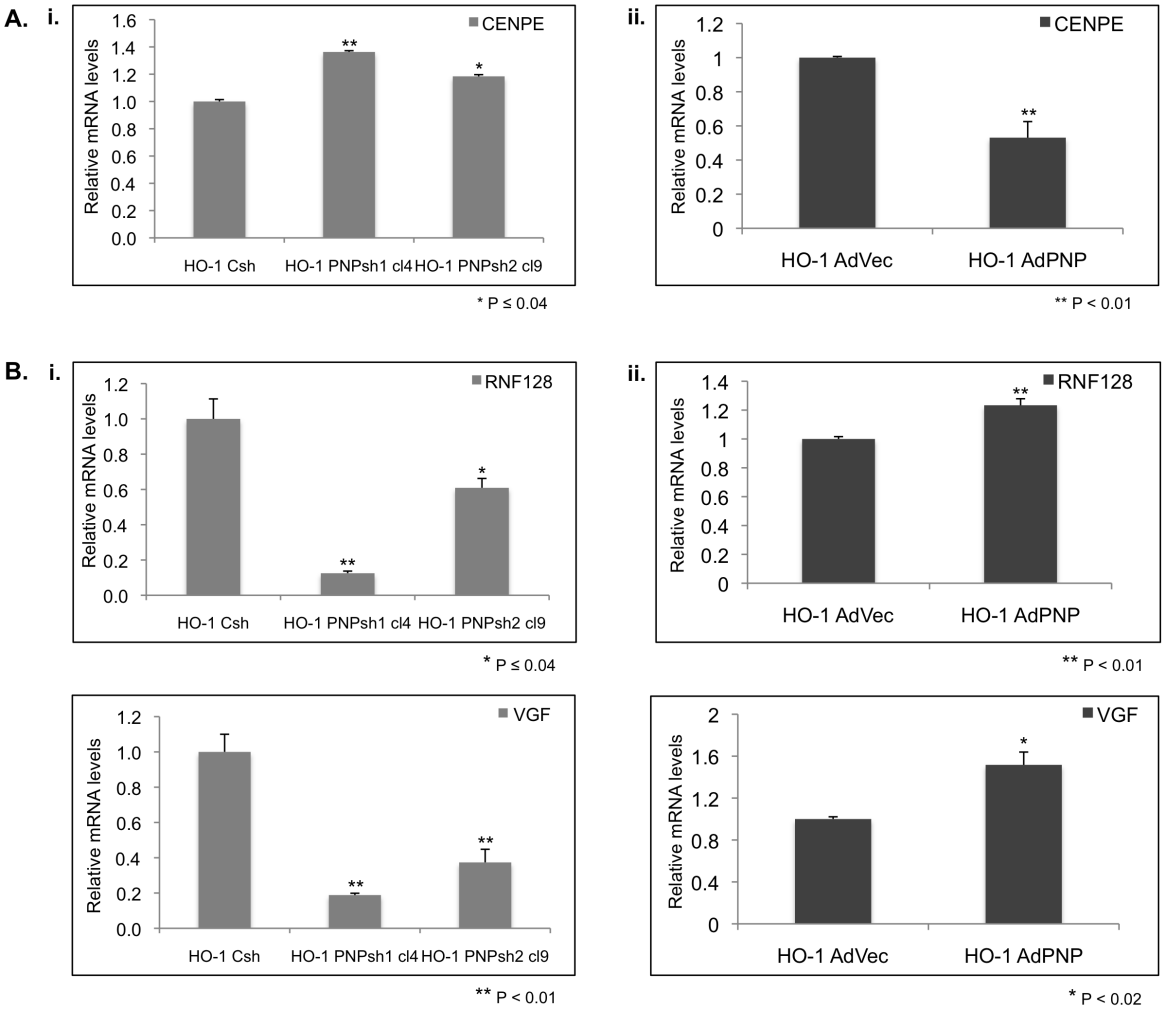
Real time qRT-PCR validation of microarray findings. (A) qRT-PCR verification of *hPNPase^old-35^*-putative “*directly*” regulated genes identified by microarray analyses in response to *hPNPase^old-35^* (i) knockdown or (ii) overexpression in HO-1 melanoma cells. (B) qRT-PCR verification of *hPNPase^old-35^*-putative “*indirectly*” regulated genes identified by microarray analyses in response to *hPNPase^old-35^* (i) knockdown or (ii) overexpression in HO-1 melanoma cells. Error bars represent mean ± S.E. error of three replicate experiments.

In order to confirm select genes from the microarray analyses (microarray fold changes provided in Table S3), five potential *hPNPase^old-35^*-regulated genes were validated (for the purpose of this study we validated only a few genes as it was not feasible to validate all the *hPNPase^old-35^*-regulated genes identified) by qRT-PCR to show their inverse correlation with *hPNPase^old-35^* expression in the HO-1 melanoma cell line as shown in Figure 9 (putative *“direct target”*: *CENPE*; putative *“indirect targets”*: *VGF*, *RNF128*). *CENPE* was chosen for validation as it was also identified in an overlapping screen we performed between *hPNPase^old-35^*-knockdown cells and a doxycycline inducible *hPNPase^old-35^* overexpression system in HeLa cells (data not shown). *VGF* and *RNF128* were chosen as they showed the maximum fold change in the *hPNPase^old-35^*-knockdown cells. The expression changes of four putative *hPNPase^old-35^*-regulated genes were also validated using another melanoma cell line WM35, in which *hPNPase^old-35^* was stably knocked down using PNPsh1 (Figures S3, S4 and S5). Transient knockdown of *hPNPase^old-35^* using siRNA (distinct from PNPsh1 and 2) also showed the inverse correlation of *hPNPase^old-35^* expression with four putative *hPNPase^old-35^*-regulated genes (Figure S6) in HO-1 melanoma cells (after 48 h). This trend was also observed in three other melanoma cell lines, WM35, C8161 and MeWo, after silencing *hPNPase^old-35^* transiently (Figures S7, S8 and S9).

## Discussion

Numerous studies performed over the past decade have utilized the RNAi methodology to evaluate the functions of *hPNPase^old-35^*. Most of these studies concentrated on studying the role of *hPNPase^old-35^* in reference to its mitochondrial location with special emphasis on mtRNA processing, maintenance of mitochondrial homeostasis and more recently its role in mitochondrial RNA import [9], [23]–[27]. In other studies, overexpression of *hPNPase^old-35^* has been shown to cause growth inhibition attributed to downregulation of c-myc and miR-221 by its exoribonuclease activity in the cytosol [11], [12]. In this study we have incorporated both these classic genetic approaches of gene knockdown and overexpression to further understand *hPNPase^old-35^* functions on a more comprehensive level. This is the first attempt of its kind aimed at studying global gene expression changes resulting from *hPNPase^old-35^* knockdown or overexpression in order to identify unique genes regulated by *hPNPase^old-35^*. In this manuscript we focus on the analysis of gene expression patterns of the most relevant biological pathways of *hPNPase^old-35^* potential functions.

Our results show that stable knockdown of *hPNPase^old-35^* in melanoma cells affects mitochondrial function and cholesterol biosynthesis. Other groups have previously reported the importance of *hPNPase^old-35^* in the maintenance of mitochondrial homeostasis through knockdown and liver specific knockout experiments where they showed a deregulation of the various respiratory complexes in the Electron Transport Chain (ETC) following *hPNPase^old-35^* silencing [9], [23]. These results further strengthen those findings. In this study we report novel genes (Table 2) that encode for proteins constituting the various ETC respiratory complexes, which were differentially expressed in our *hPNPase^old-35^* knockdown melanoma cells. Some mitochondrial accessory factors (Table 3) were also affected and one of them *UCP2* [43], which is a mitochondrial anion transporter that plays a role in energy dissipation and maintenance of mitochondrial membrane potential was downregulated ∼3-fold (Figure S2B). The exact mechanism of how *hPNPase^old-35^* regulates these genes remains to be elucidated and the possibilities may be numerous ranging from RNA degradation, miRNA regulation or RNA import. Moreover, recently mutations in *hPNPase^old-35^* have also been identified that impair respiratory-chain activity [44]. The ETC genes identified in this study identify the specific genes affected by *hPNPase^old-35^* that maybe causative for mitochondrial dysfunction. Further studies aimed at understanding the functional implications of the identified genes may provide valuable insight to the physiological role of *hPNPase^old-35^* in mitochondrial diseases and discovering suitable therapeutic options.

We also identified genes belonging to the cholesterol biosynthesis pathway that were significantly downregulated with depletion of *hPNPase^old-35^*. This is a novel and potentially biologically important result since association of *hPNPase^old-35^* with this pathway has not been previously reported. Mitochondrial dynamics have been linked to steroid biosynthesis and the gene expression changes we observed pertaining to cholesterol biosynthesis could be a consequence of mitochondrial dysfunction, or vice versa. Additionally, since this analysis was performed in a stable cell line in which *hPNPase^old-35^* was silenced, certain compensatory changes could be acquired over time as opposed to primary effects of gene knockdown. On the other hand *hPNPase^old-35^* could indeed directly regulate the RNA levels of these genes or alter their expression by regulating upstream transcriptional control elements. *SREBF2* is one such transcription factor that was found to be downregulated 1.3-fold in our microarray analyses which regulates the expression of *HMGCR* (Figure S2A), *HMGCS1* and *IDI1*. Further studies to address and evaluate these possibilities would be required to assess the relevance of *hPNPase^old-35^* in this context.

Previous studies have shown that adenoviral overexpression of *hPNPase^old-35^* induces growth suppression and a senescence-like phenotype [7], [10]. Our present analyses show that the majority of genes differentially expressed as a result of adenoviral overexpression of *hPNPase^old-35^* compared to the empty vector control (Ad.*Vec*) belong to cell cycle, cellular assembly and organization categories. Biological pathways likely affected as a consequence of the gene expression changes included cell cycle control of chromosomal replication. Key genes down-regulated more than 1.5-fold in this category belonged to the mini-chromosome maintenance complex (*MCM2*, *MCM4-7*) that is essential for eukaryotic DNA replication. Important regulators of G2/M DNA damage checkpoint control; *CCNB2*, *CHEK2* and *CDK1* were also downregulated. The gene expression changes of these cell-cycle regulators may contribute towards tipping the balance in the cell in the direction of growth inhibition. Also, changes in gene expression were identified in apoptosis regulatory molecules. *FAS*, *BIK* and *BCL2L11* (pro-apoptotic) expression was increased and *BIRC5* (anti-apoptotic) levels were reduced significantly, which may have played a role in decreasing the transmembrane potential of the mitochondrial membrane observed upon *hPNPase^old-35^* overexpression in the IPA analyses, an important phenomenon implicated in apoptosis [45]. These findings provide new information regarding the cell-cycle associated implications of *hPNPase^old-35^* overexpression and provide targets for further studies. These findings are also supported by previous studies; where it was shown that terminal differentiation (which shares overlapping characteristics with cellular senescence) of HO-1 melanoma cells caused by combination treatment with IFN-β plus the protein kinase C activator mezerein resulted in global gene expression reductions of cell-cycle associated genes [46], [47]. *hPNPase^old-35^* is a Type I-IFN inducible early response gene [8]; thus it was not surprising that we observed a similar pattern of gene expression changes in our present study involving *hPNPase^old-35^* overexpression. The candidate genes identified may also provide insight regarding the role of *hPNPase^old-35^* in aging-associated inflammation [21], [22]. Some of the potentially biologically significant genes in this category were *CAPN3*, *BIRC5*, *TRIM2*, *CENPF*, *FN1*, *BCL6* and *GMNN*. This premise needs further attention, as it is evident that the cell-cycle changes associated with *hPNPase^old-35^* overexpression are diverse and complex. Also, c-myc is a key regulator of cell-cycle progression, which is targeted by *hPNPase^old-35^* for degradation. Bearing this in mind, in the present study we chose an earlier time point of 36 hours, before changes in the *c-myc* transcript levels were evident (Figure S10B), to study Ad.*hPNPase^old-35^* associated gene expression changes. Even so, in order to identify genes directly targeted by hPNPase^old-35^ irrespective of *c-myc* status we plan to study the effects of *hPNPase^old-35^* overexpression in a *c-myc* null background [48], [49].

To identify additional genes regulated by *hPNPase^old-35^*, we ascertained transcripts in our two separate datasets (i.e. *hPNPase^old-35^* knockdown and overexpression) whose expression inversely correlated with *hPNPase^old-35^*. This novel strategy helped us detect 77 potential “*directly*” (mRNA degraded by *hPNPase^old-35^*) and 61 potential “*indirectly*” (miRNAs targeting these transcripts degraded by *hPNPase^old-35^*) regulated transcripts. Although in this study we have concentrated on this small list of genes identified through the overlapping approach between our *hPNPase^old-35^* depleted and overexpression datasets, future studies would be required to analyze the remaining genes whose expression is also regulated by *hPNPase^old-35^* but do not fall in this list (Figures 2B-C). Most of these potential “*direct*” targets were genes implicated in cell-cycle associated functions and two of them have been validated by qRT-PCR; *CENPE* (Centromere protein E) which is involved in mitotic checkpoint control [50], [51] and *MKI67* (antigen identified by monoclonal antibody Ki-67), a known cellular proliferation marker implicated in rRNA synthesis [52]-[54]. Since most of the genes thought to be “*directly*” regulated belong to a closely associated network of cell cycle regulatory functions, at this point it is difficult to say whether these gene expression changes represent global effects of *hPNPase^old-35^* deregulation caused by key upstream regulators or if they are genes that are directly targeted by *hPNPase^old-35^* for degradation. Future studies using *in vitro* mRNA degradation assays will help answer these questions. Among the “*indirectly*” regulated targets, a fraction of genes were identified that had conserved sites for miR-124a (Table S4). We validated one of these genes by qRT-PCR; *RNF128* (ring finger protein 128) an E3 ubiquitin ligase that is involved in the regulation of cytokine gene transcription [55]. Another potential indirect target we validated was *VGF* (VGF nerve growth factor inducible), which encodes a neuro-endocrine polypeptide implicated in a vast array of biological phenomena that include energy metabolism and inflammation [56]-[58]. Identification and subsequent validation of miRNAs targeting the genes we identified would aid in clarifying the role of *hPNPase^old-35^*, if any, in the context of the relevant biological processes these genes are implicated in and would further allow appropriate therapeutic intervention.

In summation, this study has produced a novel genetic and genomic analysis of the functional implications for alterations in PNPT1 gene expression. Our analysis has identified a limited set of candidate genes for direct regulation by *hPNPase^old-35^*. Such genes could provide novel targets for intervention in *hPNPase^old-35^*-related disease states. Furthermore, this work has generated a comprehensive database of *hPNPase^old-35^*-responsive genes that are potentially relevant to the mechanisms of global cellular functions affected by this important regulatory molecule.

## Supporting Information

Figure S1 **Canonical pathways associated with genes differentially expressed when** ***hPNPase^old-35^*** **is knocked down (A) or overexpressed (B) in human melanoma cells.**
(TIF)

Figure S2 **qRT-PCR verification of two most significant genes associated with (A) cholesterol biosynthesis and (B) mitochondrial dysfunction in** ***hPNPase^old-35^*** **silenced HO-1 cells.** Error bars represent mean ± S.E. of three replicate experiments.
(TIF)

Figure S3 **Stable shRNA mediated knockdown and overexpression of** ***hPNPase^old-35^*** **in WM35 melanoma cells.** (A) Phase contrast LM (top) and GFP fluorescent micrographs (bottom) of WM35 melanoma cells following transduction with GFP expressing scrambled shRNA and *hPNPase^old-35^* shRNA1 expressing lentiviruses and selection with puromycin. qRT-PCR expression of *hPNPase^old-35^* (*hPNPase^old-35^* knockdown) normalized to control (shScramble). Mean values normalized to a GAPDH internal reference; error bars represent mean ± S.E. of three replicate experiments. Anti-hPNPase^old-35^ and EF1α loading control immunoblots. (B) qRT-PCR expression of *hPNPase^old-35^* in WM35 cells infected with Ad.*hPNPase^old-35^* normalized to cells infected with Ad.*Vec* for 36 h. Immunoblot showing hPNPase^old-35^ overexpression compared to Ad.*Vec* post 36 h of infection. Error bars represent mean ± S.E. of three replicate experiments. * P<0.02, ** P<0.01.
(TIF)

Figure S4 **Real time qRT-PCR validation of** ***hPNPase^old-35^*** **-putative** ***“directly”*** **regulated genes.** qRT-PCR verification of *hPNPase^old-35^*-putative *“directly”* regulated genes identified by microarray analyses in response to *hPNPase^old-35^* (A) knockdown or (B) overexpression in WM35 melanoma cells. Error bars represent mean ± S.E. of two replicate experiments done in triplicate.
(TIF)

Figure S5 **Real time qRT-PCR validation of** ***hPNPase^old-35^*** **-putative** ***“indirectly”*** **regulated genes.** qRT-PCR verification of *hPNPase^old-35^*-putative *“indirectly”* regulated genes identified by microarray analyses in response to *hPNPase^old-35^* (A) knockdown or (B) overexpression in WM35 melanoma cells. Error bars represent mean ± S.E. of two replicate experiments done in triplicate.
(TIF)

Figure S6 **Real time qRT-PCR validation of microarray findings in HO-1 melanoma cells.** (A) qRT-PCR expression of *hPNPase^old-35^* following transient transfection with siRNA against *hPNPase^old-35^* normalized to scrambled control post 48 h in HO-1 melanoma cells. Immunoblot showing *hPNPase^old-35^* levels after siRNA transfection. (B) qRT-PCR verification of *hPNPase^old-35^*-putative (i) *“directly”* and (ii) *“indirectly”* regulated genes after *hPNPase^old-35^* transient silencing post 48 h. Error bars represent mean ± S.E. of two replicate experiments.
(TIF)

Figure S7 **Real time qRT-PCR validation of microarray findings in WM35 melanoma cells.** (A) qRT-PCR expression of *hPNPase^old-35^* following transient transfection with siRNA against *hPNPase^old-35^* normalized to scrambled control post 48 h in WM35 melanoma cells. (B) qRT-PCR verification of *hPNPase^old-35^*-putative regulated genes after *hPNPase^old-35^* transient silencing post 48 h. Error bars represent mean ± S.E. of two replicate experiments.
(TIF)

Figure S8 **Real time qRT-PCR validation of microarray findings in C8161 melanoma cells.** (A) qRT-PCR expression of *hPNPase^old-35^* following transient transfection with siRNA against *hPNPase^old-35^* normalized to scrambled control post 48 h in C8161 melanoma cells. Immunoblot showing *hPNPase^old-35^* levels after siRNA transfection. (B) qRT-PCR verification of *hPNPase^old-35^*-putative regulated genes after *hPNPase^old-35^* transient silencing post 48 h. Error bars represent mean ± S.E. of two replicate experiments.
(TIF)

Figure S9 **Real time qRT-PCR validation of microarray findings in MeWo melanoma cells.** (A) qRT-PCR expression of *hPNPase^old-35^* following transient transfection with siRNA against *hPNPase^old-35^* normalized to scrambled control post 48 h in MeWo melanoma cells. Immunoblot showing *hPNPase^old-35^* levels after siRNA transfection. (B) qRT-PCR verification of *hPNPase^old-35^*-putative regulated genes after *hPNPase^old-35^* transient silencing post 48 h. Error bars represent mean ± S.E. of two replicate experiments.
(TIF)

Figure S10 **Effect of** ***hPNPase^old-35^*** **depletion or overexpression on** ***c-myc*** **mRNA levels.** (A) qRT-PCR expression of *c-myc* following *hPNPase^old-35^* stable knockdown in HO-1 melanoma cells as identified in microarray analysis. (B) qRT-PCR expression of *c-myc* following Ad.*hPNPase^old^* infection post 36 h. Error bars represent mean ± S.E. of three replicate experiments.
(TIF)

Table S1 **Results for IPA biological functions (A), toxicity lists (B), canonical pathways (C) and most significant networks (D) associated with genes dysregulated as a result of** ***hPNPase^old-35^*** **depletion.**
(XLSX)

Table S2 **Results for IPA biological functions (A), toxicity lists (B), canonical pathways (C) and most significant networks (D) associated with genes dysregulated as a result of** ***hPNPase^old-35^*** **overexpression.**
(XLSX)

Table S3 **Potential** ***hPNPase^old-35^*** **regulated genes.** Genes significantly regulated putatively by *hPNPase^old-35^ “directly”* (A) & (B) and *“indirectly”* (C) & (D).
(XLSX)

Table S4 **Functional classification of** ***hPNPase^old-35^*** **putative** ***“directly”*** **(A) and putative** ***“indirectly”*** **(B) regulated genes by ToppGene suite.**
(XLSX)
