# Supplementary figures and images for: Identification of Genes Potentially Regulated by Human Polynucleotide Phosphorylase (hPNPaseold-35) Using Melanoma as a Model
Source: PLoS One. 2013 Oct 15;8(10):e76284. doi: 10.1371/journal.pone.0076284 (PMC3797080; doi:10.1371/journal.pone.0076284)

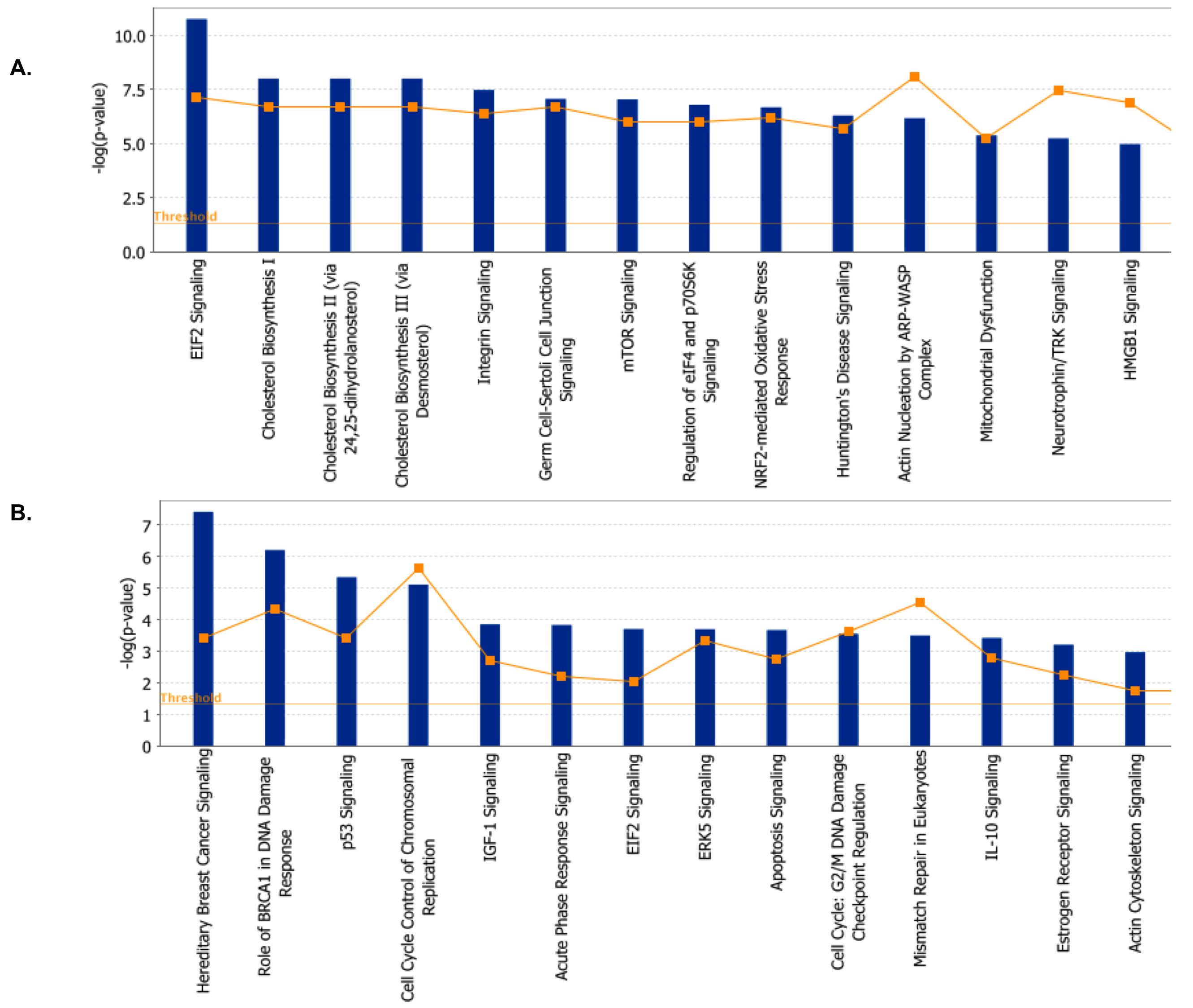

Supplement: Figure S1 — Canonical pathways associated with genes differentially expressed when hPNPaseold-35 is knocked down (A) or overexpressed (B) in human melanoma cells. (TIF) [file pone.0076284.s001.tif]

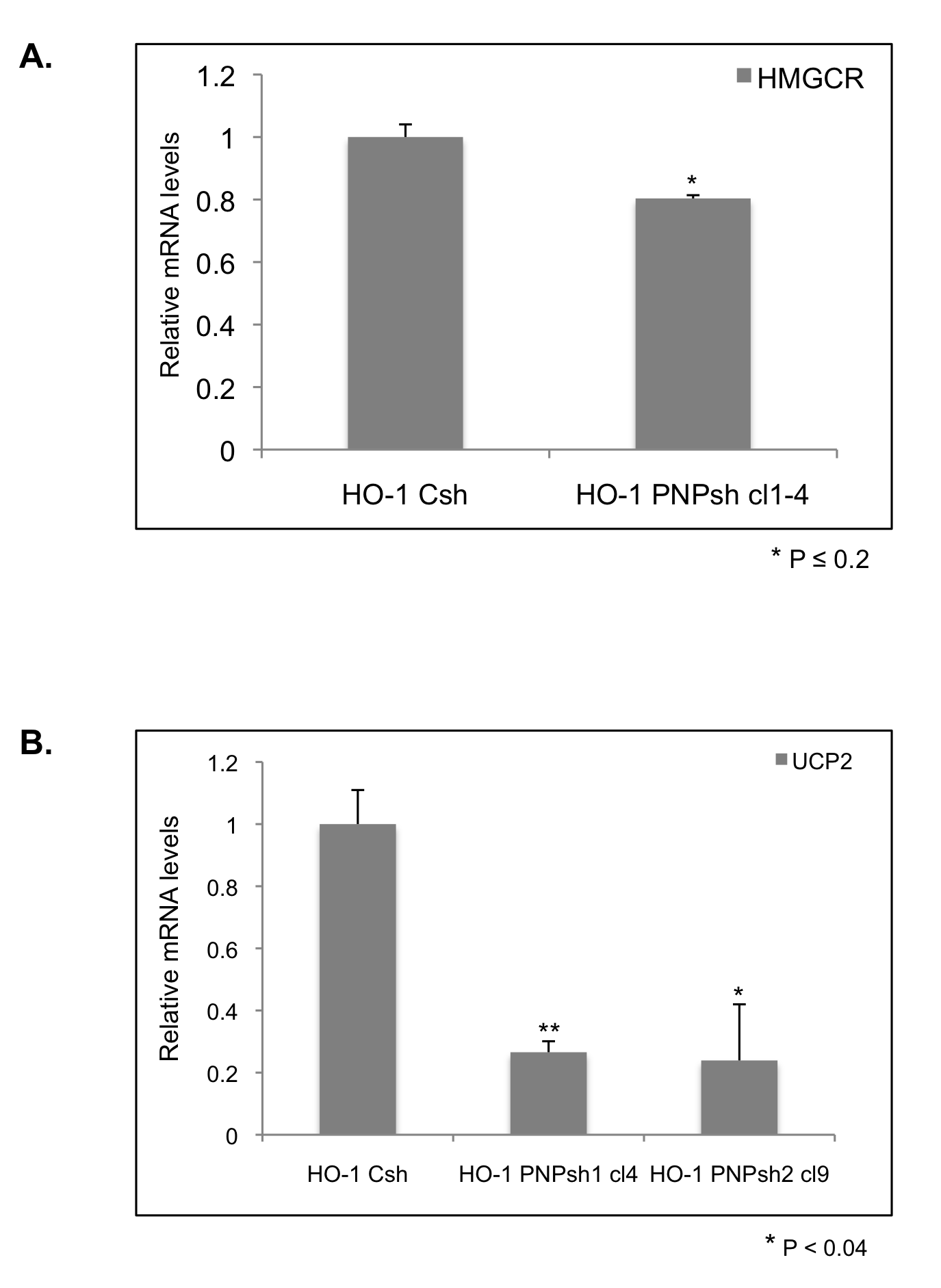

Supplement: Figure S2 — qRT-PCR verification of two most significant genes associated with (A) cholesterol biosynthesis and (B) mitochondrial dysfunction in hPNPaseold-35 silenced HO-1 cells. Error bars represent mean ± S.E. of three replicate experiments. (TIF) [file pone.0076284.s002.tif]

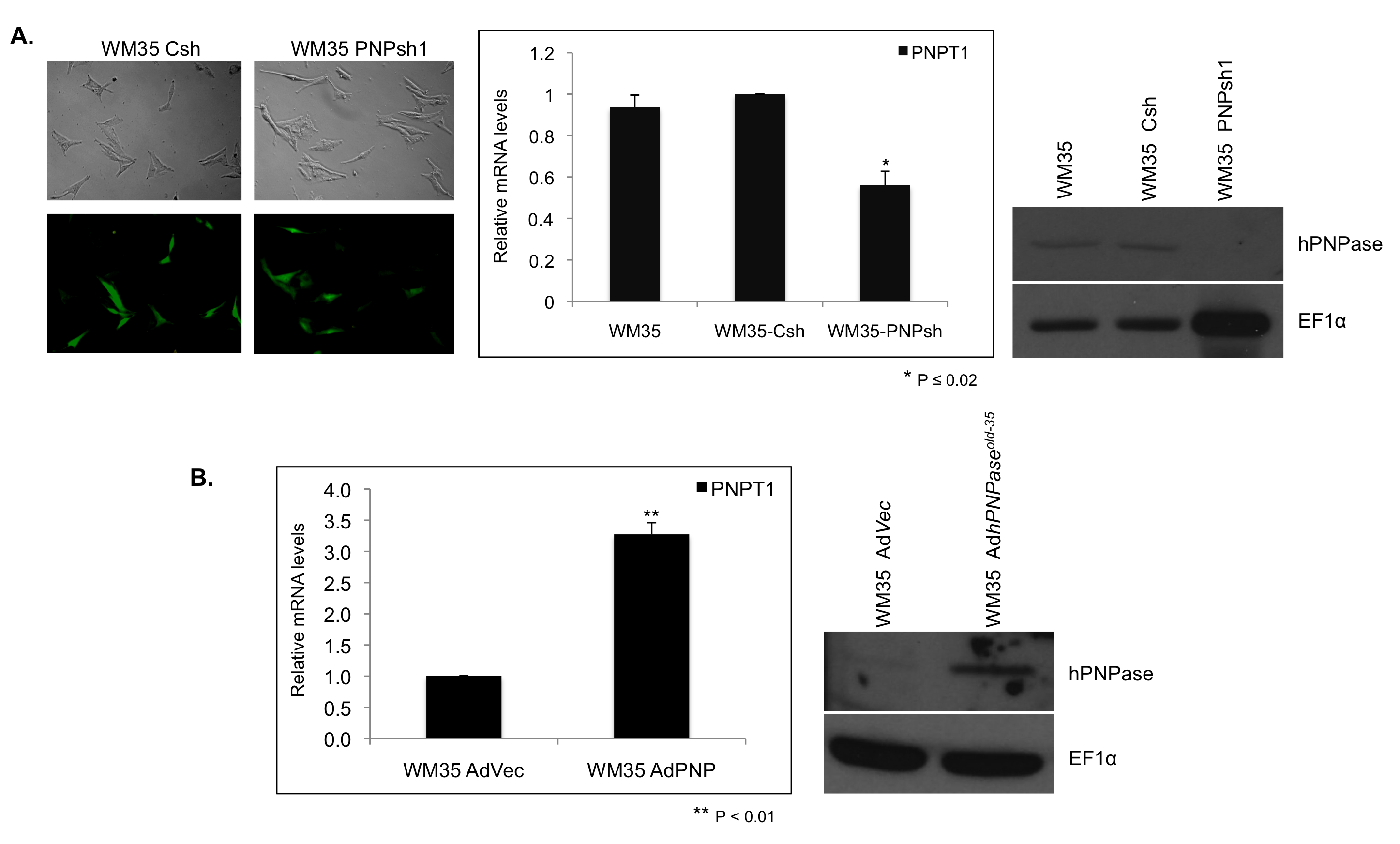

Supplement: Figure S3 — Stable shRNA mediated knockdown and overexpression of hPNPaseold-35 in WM35 melanoma cells. (A) Phase contrast LM (top) and GFP fluorescent micrographs (bottom) of WM35 melanoma cells following transduction with GFP expressing scrambled shRNA and hPNPaseold-35 shRNA1 expressing lentiviruses and selection with puromycin. qRT-PCR expression of hPNPaseold-35 (hPNPaseold-35 knockdown) normalized to control (shScramble). Mean values normalized to a GAPDH internal reference; error bars represent mean ± S.E. of three replicate experiments. Anti-hPNPaseold-35 and EF1α loading control immunoblots. (B) qRT-PCR expression of hPNPaseold-35 in WM35 cells infected with Ad.hPNPaseold-35 normalized to cells infected with Ad.Vec for 36 h. Immunoblot showing hPNPaseold-35 overexpression compared to Ad.Vec post 36 h of infection. Error bars represent mean ± S.E. of three replicate experiments. * P<0.02, ** P<0.01. (TIF) [file pone.0076284.s003.tif]

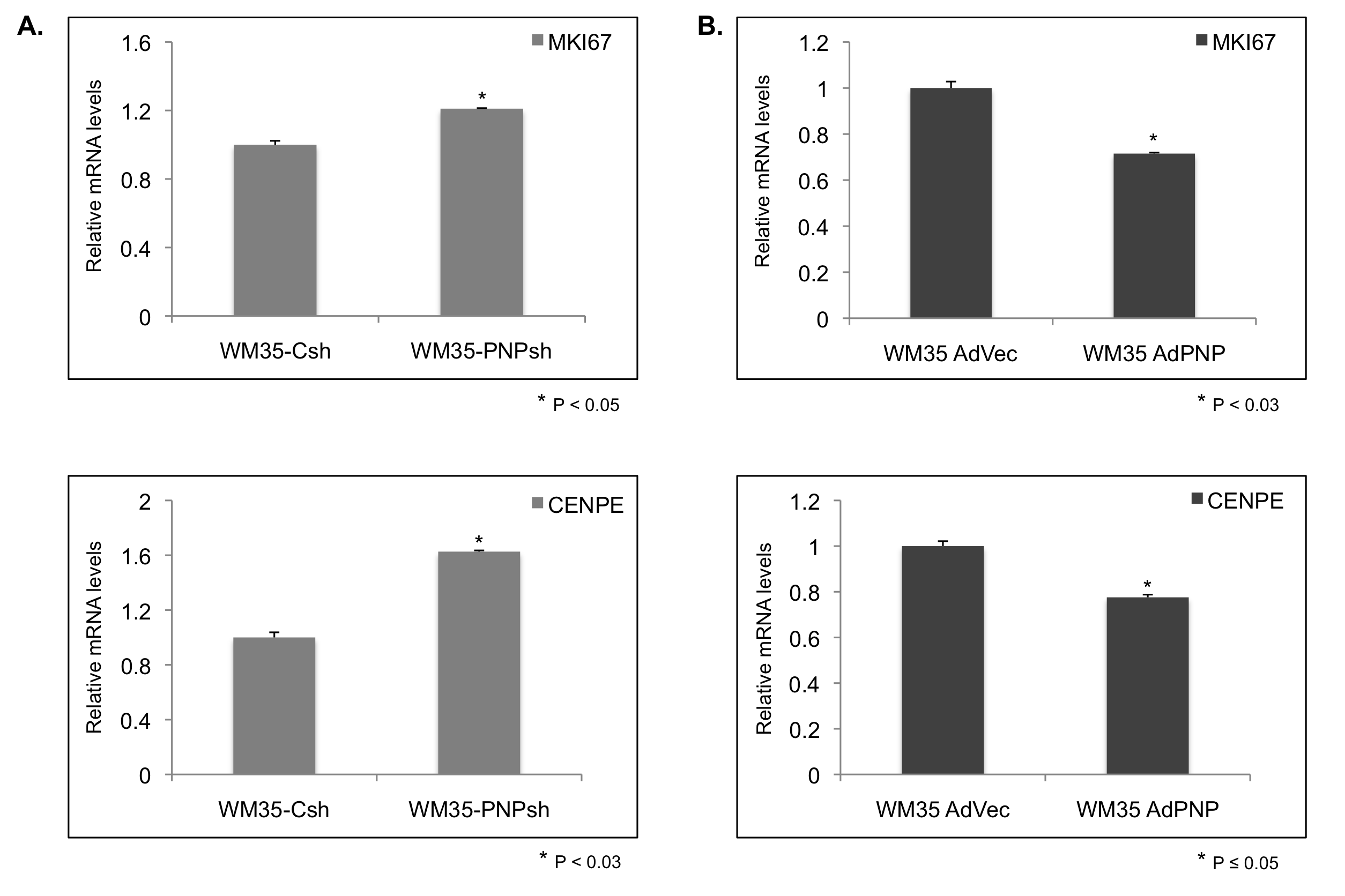

Supplement: Figure S4 — Real time qRT-PCR validation of hPNPaseold-35 -putative “directly” regulated genes. qRT-PCR verification of hPNPaseold-35-putative “directly” regulated genes identified by microarray analyses in response to hPNPaseold-35 (A) knockdown or (B) overexpression in WM35 melanoma cells. Error bars represent mean ± S.E. of two replicate experiments done in triplicate. (TIF) [file pone.0076284.s004.tif]

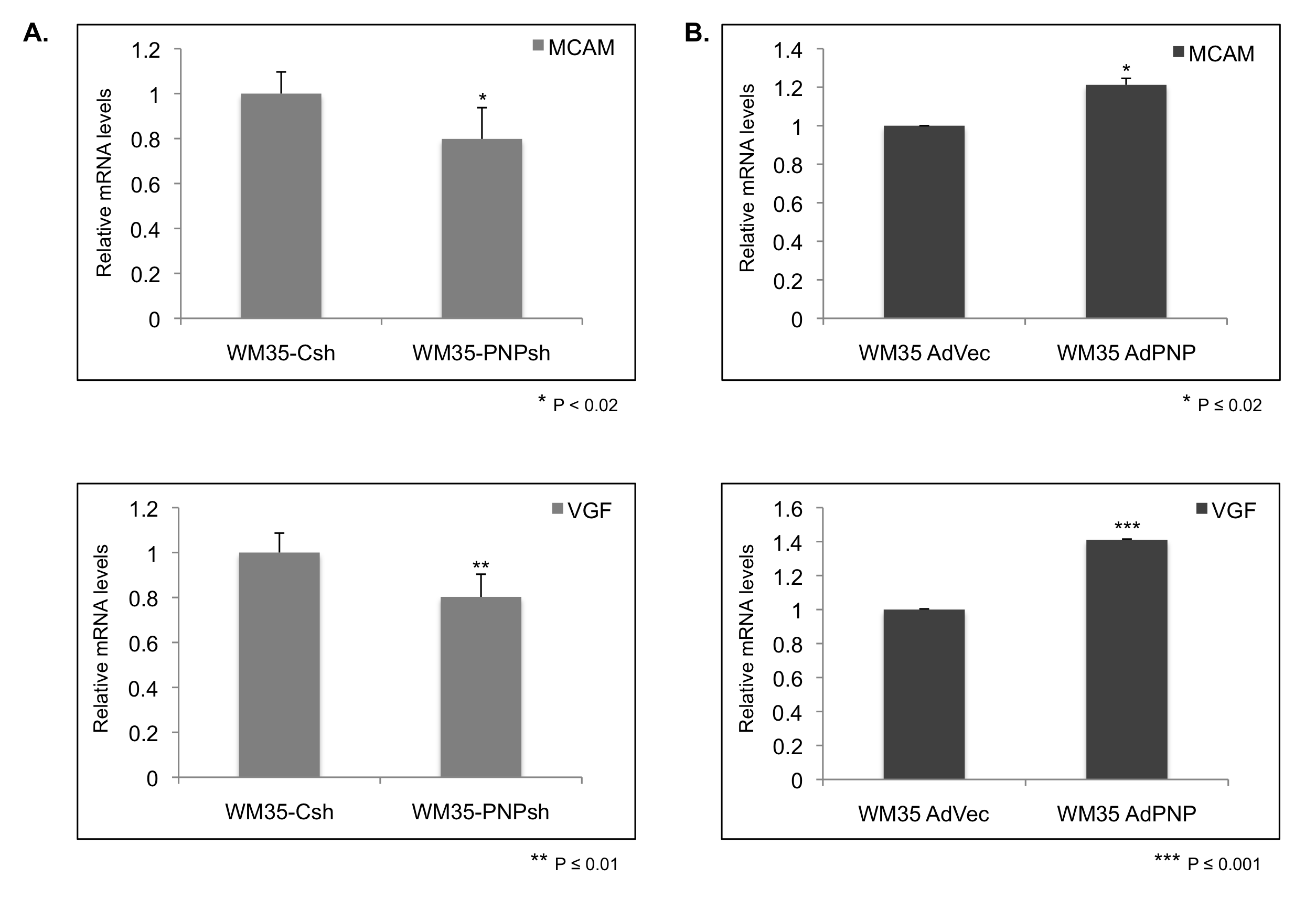

Supplement: Figure S5 — Real time qRT-PCR validation of hPNPaseold-35 -putative “indirectly” regulated genes. qRT-PCR verification of hPNPaseold-35-putative “indirectly” regulated genes identified by microarray analyses in response to hPNPaseold-35 (A) knockdown or (B) overexpression in WM35 melanoma cells. Error bars represent mean ± S.E. of two replicate experiments done in triplicate. (TIF) [file pone.0076284.s005.tif]

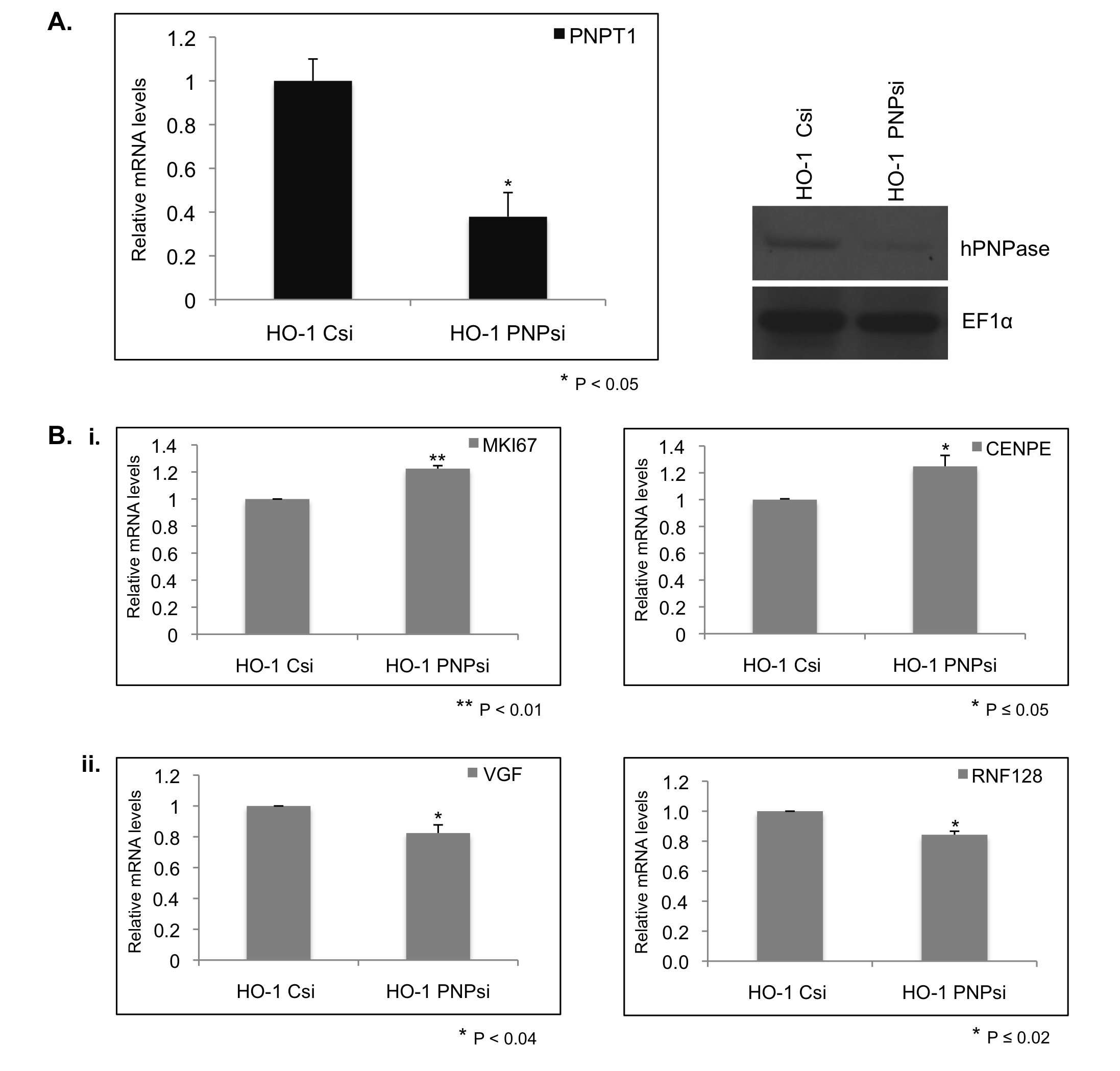

Supplement: Figure S6 — Real time qRT-PCR validation of microarray findings in HO-1 melanoma cells. (A) qRT-PCR expression of hPNPaseold-35 following transient transfection with siRNA against hPNPaseold-35 normalized to scrambled control post 48 h in HO-1 melanoma cells. Immunoblot showing hPNPaseold-35 levels after siRNA transfection. (B) qRT-PCR verification of hPNPaseold-35-putative (i) “directly” and (ii) “indirectly” regulated genes after hPNPaseold-35 transient silencing post 48 h. Error bars represent mean ± S.E. of two replicate experiments. (TIF) [file pone.0076284.s006.tif]

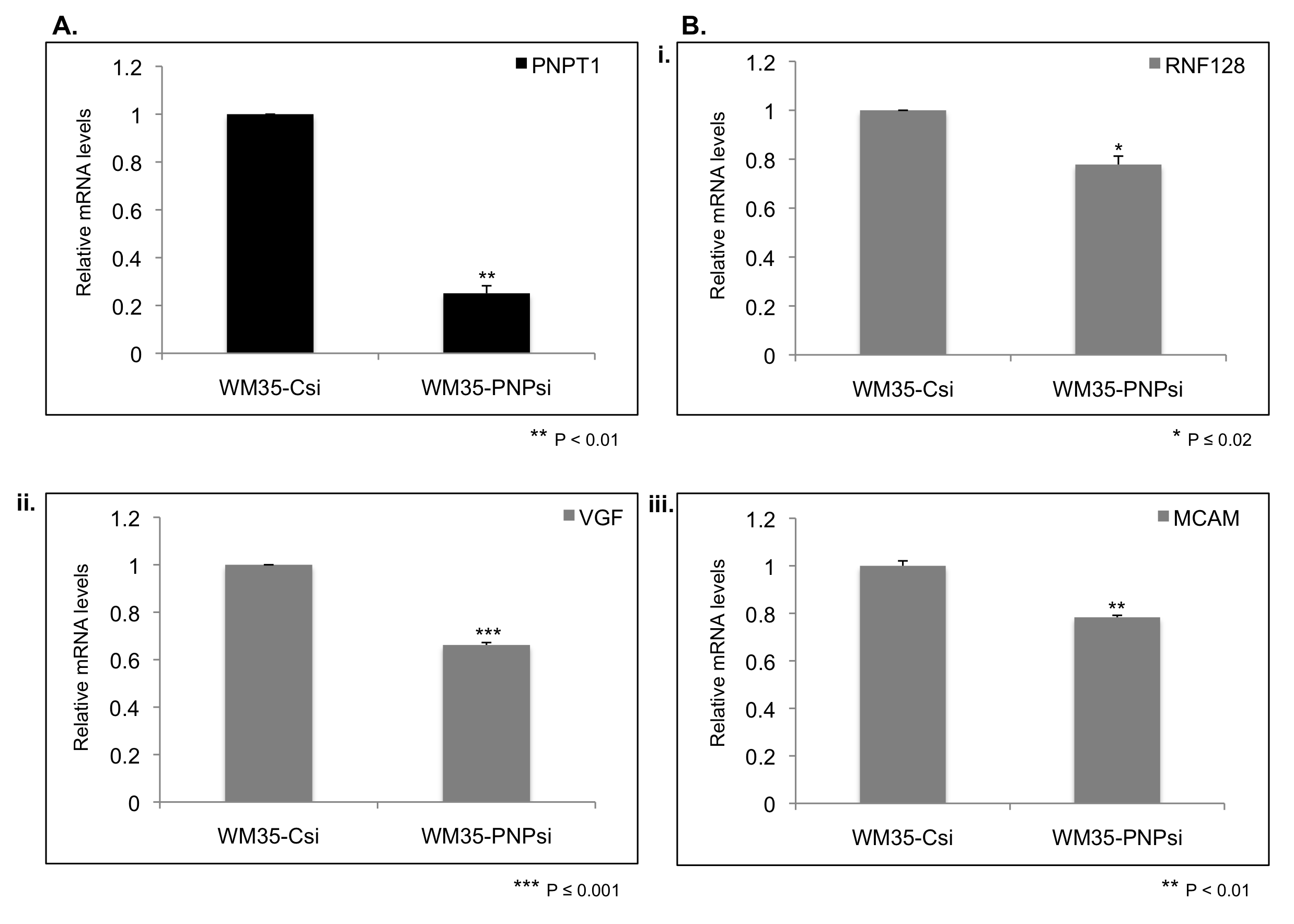

Supplement: Figure S7 — Real time qRT-PCR validation of microarray findings in WM35 melanoma cells. (A) qRT-PCR expression of hPNPaseold-35 following transient transfection with siRNA against hPNPaseold-35 normalized to scrambled control post 48 h in WM35 melanoma cells. (B) qRT-PCR verification of hPNPaseold-35-putative regulated genes after hPNPaseold-35 transient silencing post 48 h. Error bars represent mean ± S.E. of two replicate experiments. (TIF) [file pone.0076284.s007.tif]

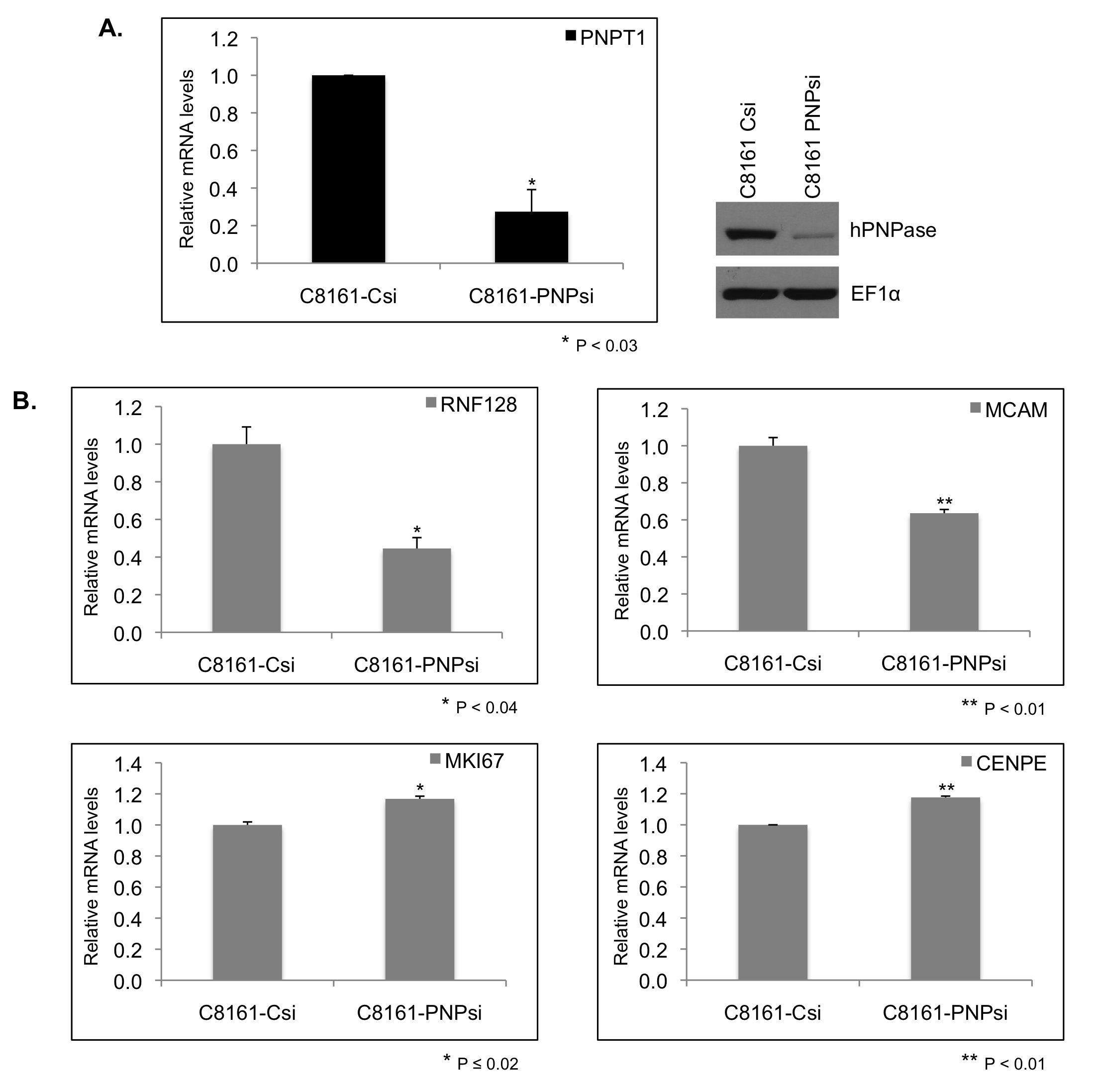

Supplement: Figure S8 — Real time qRT-PCR validation of microarray findings in C8161 melanoma cells. (A) qRT-PCR expression of hPNPaseold-35 following transient transfection with siRNA against hPNPaseold-35 normalized to scrambled control post 48 h in C8161 melanoma cells. Immunoblot showing hPNPaseold-35 levels after siRNA transfection. (B) qRT-PCR verification of hPNPaseold-35-putative regulated genes after hPNPaseold-35 transient silencing post 48 h. Error bars represent mean ± S.E. of two replicate experiments. (TIF) [file pone.0076284.s008.tif]

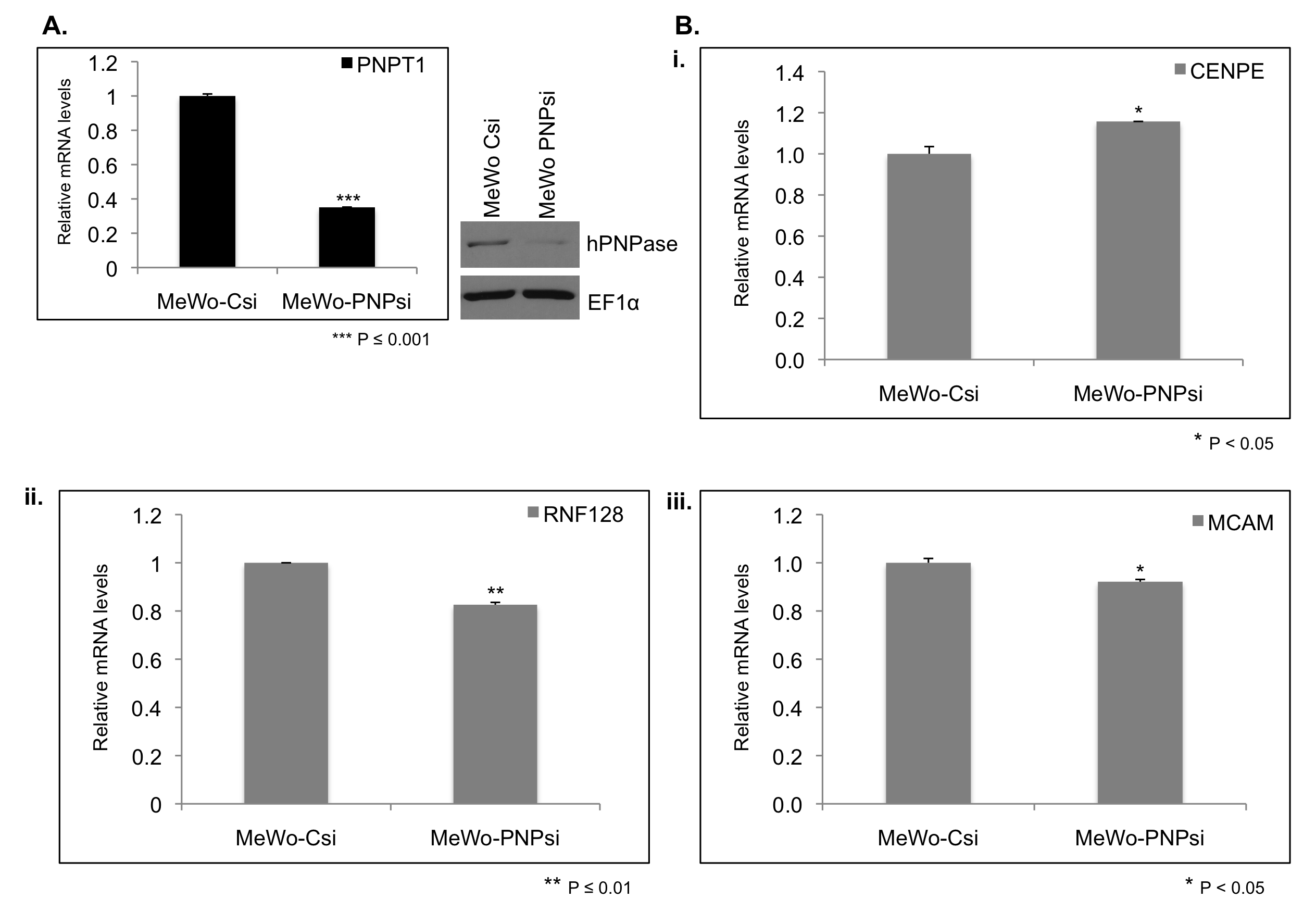

Supplement: Figure S9 — Real time qRT-PCR validation of microarray findings in MeWo melanoma cells. (A) qRT-PCR expression of hPNPaseold-35 following transient transfection with siRNA against hPNPaseold-35 normalized to scrambled control post 48 h in MeWo melanoma cells. Immunoblot showing hPNPaseold-35 levels after siRNA transfection. (B) qRT-PCR verification of hPNPaseold-35-putative regulated genes after hPNPaseold-35 transient silencing post 48 h. Error bars represent mean ± S.E. of two replicate experiments. (TIF) [file pone.0076284.s009.tif]

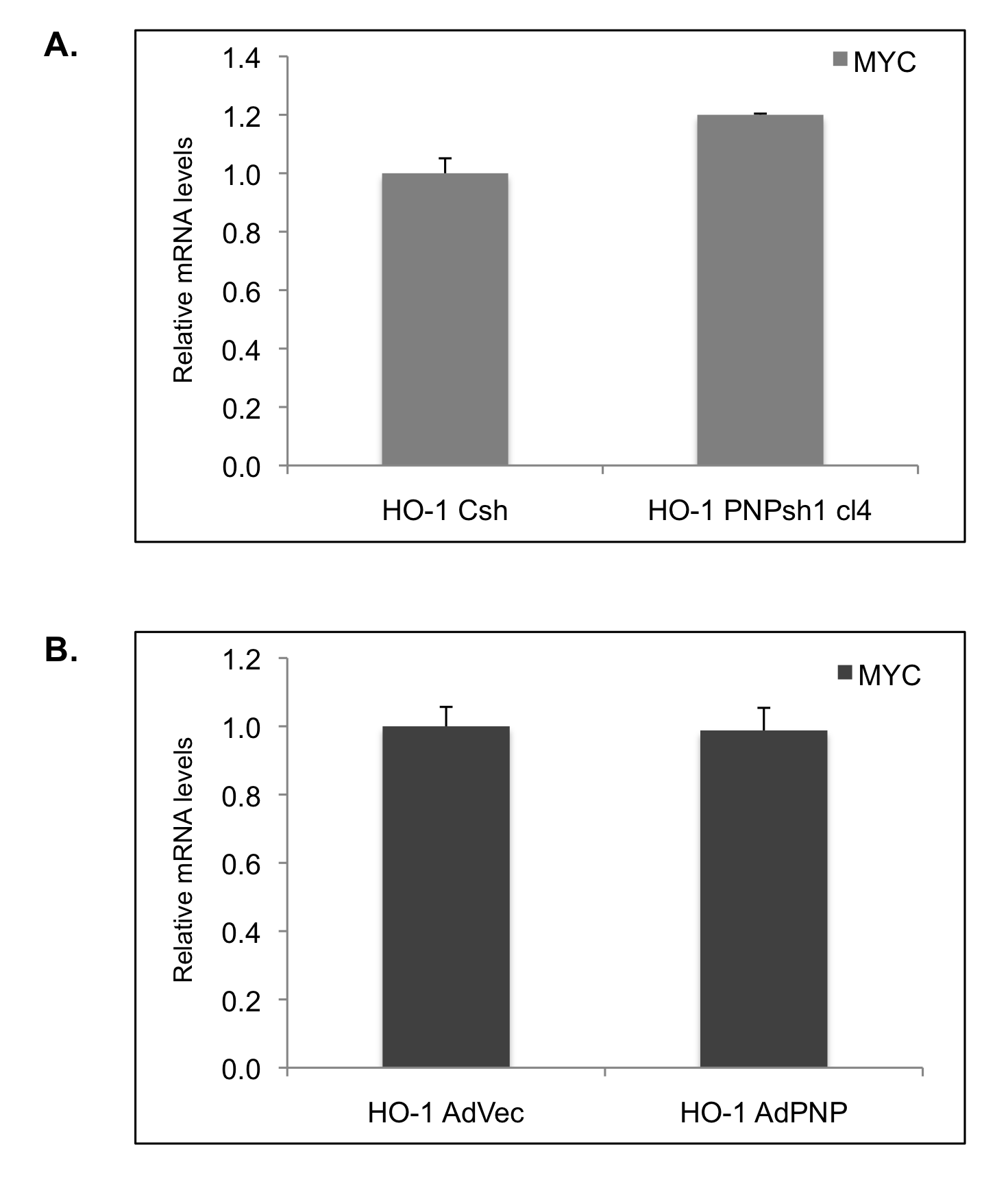

Supplement: Figure S10 — Effect of hPNPaseold-35 depletion or overexpression on c-myc mRNA levels. (A) qRT-PCR expression of c-myc following hPNPaseold-35 stable knockdown in HO-1 melanoma cells as identified in microarray analysis. (B) qRT-PCR expression of c-myc following Ad.hPNPaseold infection post 36 h. Error bars represent mean ± S.E. of three replicate experiments. (TIF) [file pone.0076284.s010.tif]
